# Perspectives on the Role of Isoliquiritigenin in Cancer

**DOI:** 10.3390/cancers13010115

**Published:** 2021-01-01

**Authors:** Kai-Lee Wang, Ying-Chun Yu, Shih-Min Hsia

**Affiliations:** 1 Department of Nursing, Ching Kuo Institute of Management and Health, Keelung 20301, Taiwan; kellywang@tmu.edu.tw; 2 School of Nutrition and Health Sciences, College of Nutrition, Taipei Medical University, Taipei 11031, Taiwan; 3 Sex Hormonal Research Center, China Medical University Hospital, Taichung 40403, Taiwan; yingchun.ycyu@gmail.com; 4 Department of Obstetrics and Gynecology, School of Medicine, China Medical University, Taichung 40403, Taiwan; 5 Graduate Institute of Metabolism and Obesity Sciences, College of Nutrition, Taipei Medical University, Taipei 11031, Taiwan; 6 School of Food and Safety, Taipei Medical University, Taipei 11031, Taiwan; 7 Nutrition Research Center, Taipei Medical University Hospital, Taipei 11031, Taiwan

**Keywords:** isoliquiritigenin, cancer, apoptosis, cell signaling

## Abstract

**Simple Summary:**

Isoliquiritigenin (ISL), a natural bioactive compound with a chalcone structure, demonstrates high antitumor efficacy. This review presents a summary of the latest research on the metabolites, pharmakinetics, and pharmacological effects of ISL and its derivatives. We highlighted the therapeutic molecular targets that are involved in antitumor effects in different cancers, both in vivo and in vitro. We also summarized the role of ISL, providing a full account of the applications of ISL thus far in various therapeutic schemes in the treatment of different cancers, alone or in combination with other drugs.

**Abstract:**

Isoliquiritigenin (2′,4′,4-trihydroxychalcone, ISL), one of the most important bioactive compounds with a chalcone structure, is derived from licorice root. Licorice is commonly known as *Glycyrrhiza*, including *Glycyrrhiza uralensis*, *Glycyrrhiza radix*, and *Glycyrrhiza glabra*, which are generally available in common foods and Chinese herbal medicines based on a wide variety of biological functions and pharmacological effects, and its derivative (ISL) is utilized as a food additive and adjunct disease treatment. In this review, we summarized the progress over the last 10 years in the targeted pathways and molecular mechanisms of ISL that are involved in the regulation of the onset and progression of different types of cancers.

## 1. Introduction

Cancer is one of the leading causes of morbidity and mortality worldwide. Based on GLOBOCAN (https://gco.iarc.fr) estimates, approximately 18 million new cancer cases and 9.6 million deaths occurred in 2018 worldwide. Nowadays, it is the second leading cause of death (9.6 million) globally [1]. Due to the changes in lifestyle habits such as smoking, overweight, physical inactivity, and reproductive patterns associated with urbanization and economic development, the global morbidity and mortality of cancer is predicted to increase quickly over the next few decades. The most common causes of cancer-related death are lung cancer, colorectal cancer, stomach cancer, liver cancer, and breast cancer. Conventional cancer therapies, including surgery, radiotherapy, and chemotherapy, are the most common strategies to combat cancer [2]. These therapies are showing more and more limitations because of their poor prognosis and side effects. However, poor prognosis occurs when cancer is resistant to radiotherapy (radioresistance) and chemotherapy (chemoresistance), which presents a challenge in cancer therapeutics. A better therapeutic strategy has the characteristics of low toxicity, high antitumor activity, and specific multi-targeting properties, avoiding the high mortality rate and decreasing the prolonged survival time for metastatic cancer to date. Seeking natural compounds from herbal remedies that possess the high efficacy and low adverse effects associated with cancer or that target cancer themselves is the ultimate cure for cancer. Without any better solution, medicinal plants act as an alternative therapy to improve the unmet needs of cancer survivors.

Licorice extracts are one of the most common Chinese herbs widely applied in traditional medicine. Licorice belongs to the genus *Glycyrrhiza*, and *Glycyrrhiza radix* is the dried roots and rhizome of licorice. Licorice had been used for diseases since the Former Han dynasty (the second–third centuries B.C.), and has been documented in ancient Egypt, Greek, and Rome. The pharmacological effects of licorice have been demonstrated for peptic ulcers, constipation, coughs, and other diseases, especially in cancer therapy. However, high doses of licorice have a risk of side effects, such as cardiac dysfunction, edema, hypertension and hypokalemic-induced secondary disorders [3]. It is necessary to identify a more potential candidate from the licorice to improve human health and lifespan. It has been known that one of the most important bioactive candidates in licorice is isoliquiritigenin (2′,4′,4-trihydroxychalcone, ISL).

ISL serves as one of the most active components in *Glycyrrhiza*. For example, Lee et al. (2013) found that ISL remarkably suppresses the receptor activator of nuclear factor kappa-B ligand (RANKL)-induced osteoclast formation of murine bone marrow-derived macrophages [4]. ISL does not only show the same pharmacologic effects as *Glycyrrhiza*, but also exerts more biological activities, especially in terms of antitumor effects. Clinical trials using ISL alone and in combination against cancer are still in their infancy. However, based on the many in vitro and in vivo studies conducted in various research laboratories across the world, the results are encouraging. In the present review, we summarize the most recent research in the literature on the phytochemical properties and pharmacological applications of ISL to provide further support and evidence for cancer treatments.

## 2. ISL’s Metabolites, Pharmakinetics, and Pharmacological Effects

### 2.1. ISL Metabolites

**Figure 1:**
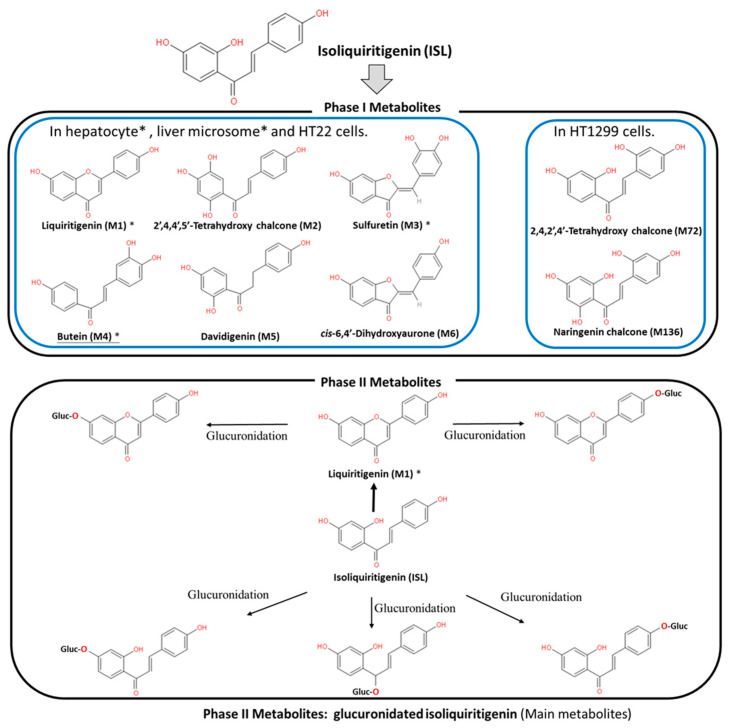
Metabolites of isoliquiritigenin (ISL). Phase I ISL metabolites were identified to be liquiritigenin (M1), 2′,4,4′,5′-tetrahydroxychalcone (M2), sulfuretin (M3), butein (M4), davidigenin (M5), and *cis*-6,4′-dihydroxyaurone (M6). Phase II metabolites were glucuronide conjugated process. Note: Figure was modified from [5,7,8].

ISL is a flavonoid with a simple chalcone structure. The structure of ISL and its metabolites are shown in Figure 1. The previous studies demonstrated the six metabolites detected in phase I [5,6,7], including liquritigenin (M1), 2′,4,4′,5′-tetrahydroxychalcone (M2), sulfuretin (M3), butein (M4), davidigenin (M5), and *cis*-6,4′-dihydroxyaurone (M6). Among the six metabolites, butein is the more active metabolite in the liver and in HT22 cells, with significant distribution on M1, M3, and M4 (Figure 1) [5,6,8]. Moreover, the previous study reported that the dominant metabolites of ISL are THC (2,4,2′,4′-tetrahydroxychalcone) and naringenin chalcone in lung cells [9]. In vivo absorption of ISL occurs in the intestines, transported to the liver for phase II biotransformation [7]. In phase II metabolism, liquiritigenin, glucuronidated ISL, glucuronidated liquiritigenin, and glucuronidated ISL are produced. Only glucuronidated liquiritigenin is predominant [10]. Many studies have suggested that secondary metabolites are involved in different biological activities and pharmaceuticals [5,7,8,11]. Therefore, these metabolites may differ in various cell lines or organs; however, they all share a similar structure to that of chalcone, which contains two aromatic rings connected by an unsaturated carbon chain, resulting in interconnected biological activities.

### 2.2. ISL Pharmacokinetics

Evaluation of the safety of ISL is necessary for future clinical applications. Therefore, many studies, through different routes of administrations, including intravenously (IV), via hypodermic (IH) or intraperitoneal (IP) injection, and orally, have indicated that ISL exhibits a robust absorption capacity (absorption rate: ~60–90 min; oral absorption: >90%) with a strong elimination ability (t_1/2_: 2–4.9 h) [10,12,13,14]. Moreover, the data showed similar trends among different analytic methods, including high-performance liquid chromatography (HPLC), HPLC–MS/MS, and fluorescence spectrometry (SFS) [10,12,13]. This means that the absorption of ISL is quickly and widely distributed throughout the body [10,12,13,14]. Concentrations of ISL may vary in different tissues, including the heart, liver, lungs, spleen, kidneys, brain, muscles, and fat. ISL distribution mainly relies on the blood circulation, with the brain showing the lowest level of ISL due to the blood–brain barrier (BBB). These results imply that ISL is able to penetrate the BBB and exhibits neuroprotective activity in a male middle cerebral artery occlusion (MCAO)-induced focal cerebral ischemia rat model and high fat diet (HFD)-induced ICR mice model [15,16]. Interestingly, only after oral administration does [ISL]_plasma_ exhibit a double-peak of ISL [14,17,18,19], the possible mechanism for which has been proposed as enterohepatic recycling. As a matter of fact, oral administration has become the most advanced application route.

### 2.3. ISL Nanoformulations and ISL Derivatives: Improved Efficacy

Generally speaking, poor bioavailability, rapid degradation, fast metabolism, and systemic elimination are the essential factors that lead to insufficient bioavailability. Insufficient bioavailability of ISL means that its efficacy is far less than 20% [10,14]. The term insufficient bioavailability implies that patients show intolerance to bulk administration of ISL to reach the desired effect, thereby highlighting the need to improve its effectiveness. To improve solubility, enhancing its bioavailability and distribution, encapsulated ISL nanoparticles or nano-ISL have been developed. Below, we summarize various ISL nanoparticles applied in preclinical studies, for example, polymer nanoparticles, liposomes, micelles, solid lipid nanoparticles (SLNs), and polymer conjugates.

1. Nanosuspension: ISL is milled with HPC (hydroxypropyl cellulose) SSL and PVP (polyvinylpyrrolidone) K30 to form a lamelliform or ellipse shape of the nanosuspension. HPC SSL and PVP K30 act as stabilizer. These two nanosuspension particles (size: 238.1 ± 4.9 nm with SSL; 354.1 ± 9.1 nm with K30) do not only improve the solubility issue, but also enhance the cytotoxicity a 7.5–10-fold [20].
2. Nanoencapsulation: Mesoporous silica nanoparticles (MSNs) are a solid material, acting as a biodegradable nanoscale drug carrier. When MSNs are encapsulated with ISL, they improve the efficacy of ISL in vitro and in vivo [21].
3. Lipid–polymer hybrid nanoparticle system:
  3.1. iRGD hybrid NPs: The composition of lipid–polymer hybrid nanoparticles (NPs) include lactic-co-glycolic acid (PLGA), lecithin, and a hydrophilic poly-ethylene-glycol (PEG). ISL-loaded hybrid NPs are composed of an inner PLGA core with an outer lipid layer (PEG, lecithin, and iRGD peptides). iRGD peptides (CRGDK/RGPD/EC, a tumor-homing peptides), can deliver drugs to a tumor. In vitro, ISL–iRGD NPs show stronger inhibition effects and induce apoptosis effects. In vivo, ISL–iRGD NPs show stronger effects in the viability of tumor cells. Herein, iRGD-modified lipid–polymer NPs showed better solubility, bioavailability, and targeting distribution [22].
  3.2. Hydrophilic polyanion solid lipid nanoparticles (SLNs): SLNs are composed of natural lipids such as lecithin or triglycerides that remain solid at 37 °C. SLNs can protect labile compounds from chemical degradation and can improve bioavailability. Low-molecular-weight heparins (LMWHs) are fragments of heparin showing hydrophilic polyanions that can improve the efficacy of ISL [23].
4. Microemulsion: The self-microemulsifying drug delivery system (SEMDDS) was designed for improving the solubility, absorption, and bioavailability of lipophilic drugs. The SMEDDS comprises ethyl oleate (EO; oil phase), Tween 80 (surfactant), and PEG 400 (co-surfactant). ISL-loaded SMEDDS has been proven to improve the solubility and oral in vivo availability [17].
5. ISL-loaded nanostructured lipid carriers (ISL-NLCs): NLCs mix solid lipids with spatially incompatible liquid lipids, which leads to a special nanostructure with improved properties for drug loading. ISL-loaded NLCs are constructed by glycerol monostearate (MS) and Mi-glyol-812 as the solid and liquid lipid materials to carry the ISL [24]. In pharmacokinetic studies, less than 10% of the NLCs remains in the stomach after oral administration, mainly absorbed in the colon [19]. Moreover, the antitumor effect of ISL-loaded NLCs has been evaluated in sarcoma 180 (S180)-bearing and murine hepatoma (H22)-bearing mice models via IP administration [24]. A biodistribution study showed that the ISL concentration of ISL-loaded NLCs in the tumor is higher 2.5-fold than free ISL. In a skin permeability study, the previous study suggested NLCs as a promising carrier to deliver the ISL [25].
6. TPGS-modified proliposomes: D-α-tocopheryl polyethylene glycol 1000 succinate (TPGS) has been selected as an excipient for ISL-loaded TPGS-modified proliposomes (ISL-TPGS-PLP), prepared using the film dispersion method with ISL-loaded proliposomes (ISL–PLP). ISL-TPGS-PLP can enhance the solubility, bioavailability and liver-targeting ability of ISL [18].
7. Polymeric micelles: PEO (polyethylene oxide)–PPO (polypropylene oxide)–PEO (polyethylene oxide) triblock copolymers are highly biocompatible and act as surface-active agents. P123 (PEO20–PPO65–PEO20) can remarkably enhance the retention of poorly soluble drugs in the blood circulation. Another important derivative of Pluronic, F127 (PEO100–PPO69–PEO100), possesses high biocompatibility. Therefore, mixed F127/P123 polymeric micelles have been developed, which have remarkably enhanced bioavailability with high encapsulation efficiency and low particle size. ISL-loaded F127/P123 polymeric micelles (ISL-FPM) improve the solubility as well as enhance the bioavailability and antioxidant activity of ISL [26].
8. Nanoliposomes (NLs): Drug-loaded PEGylated nanomaterials have shown effective cancer cell-killing ability, PEG2000-DPSE-QUE-NLs (polyethyleneglycol-2000-distearoyl phosphatidyl ethanolamine loaded with querce-tin (QUE)) can efficiently disperse in aqueous media compared to controls, and PEGylated (PEG2000-DPSE) NLs have been found to be effective drug delivery vehicles when simply loaded with ISL. ISL-NLs as tumor-targeted drug carriers are more effective in regulating glycolysis in colon cancer cell lines (CRC: HCT116) [27].
9. Hydrogel: Hydrogels are composed of hyaluronic acid (HA) and hydroxyethyl cellulose (HEC), and they can improve the skin permeation of ISL [28].

As described above, many experiments have been conducted to evaluate the various properties of ISL nanoformulation have been developed to address the problems of bioavailability and solubility. Nanoformulation studies have been conducted in vitro and in vivo (Table 1), demonstrating that ISL nanoformulations improve the bioavailability by 2–10-fold [17,24,26].

**Figure 2:**
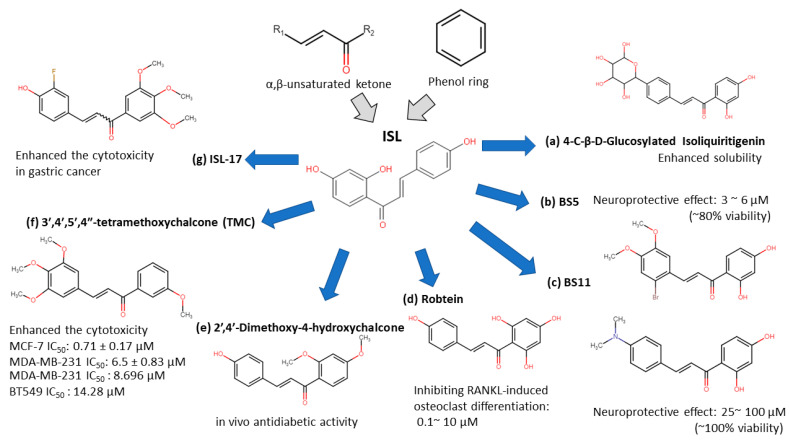
Isoliquiritigenin (ISL) derivatives.

ISL-derived new compounds offer another solution to improve the bioavailability and water-soluble issues [31,32,33,34,35,36]. Considering the chalone structure, the α,β-unsaturated ketone is an important part of its biological activity by modifying on the phenol ring to improve the performance of ISL. We summarized a few new analogues of ISL in below (see Figure 2):

1. 4-C-β-D-glucosylated ISL (Figure 2a): Glucosylation of low molecular weight compounds have improve water solubility and bioavailability with a good inhibition of aldose reductase (AR) [37].
2. Synthetic isoliquiritigenin derivatives (BS5 and BS11 in Figure 2b,c): The compounds BS5 and BS11 with m-, p-dimethoxy, o-bromo phenyl group shows neuroprotective effects at 3 μM to 6 μM with higher viability (~80–100%) [36].
3. Robtein (ISL-derivative #10; Figure 2d): Robtein exhibited osteoclast differentiation and activation without any significant changes of viability or cytotoxicity [34].
4. 2′,4′-dimethoxy-4-hydroxychalcone (Figure 2e): shows in vivo antidiabetic activity [35].
5. 3′,4′,5′,4″-tetramethoxychalcone (TMC; Figure 2f): Introducing methylation of hydroxy groups significant increase cytotoxic activity in breast cancer [31], especially targeting on triple-negative breast cancer (TNBC) [33].
6. ISL-17 (Figure 2g): A fluorine atom was introduced to the structure of ISL named ISL-17 showed the anti-tumor activities in gastric cancer [32].

However, the poor bioavailability and water-solubility issues remain in clinical applications. Future studies are still needed to elucidate the ISL formulations that would be more suitable for human clinical trials.

### 2.4. ISL Docking Model

**Figure 3:**
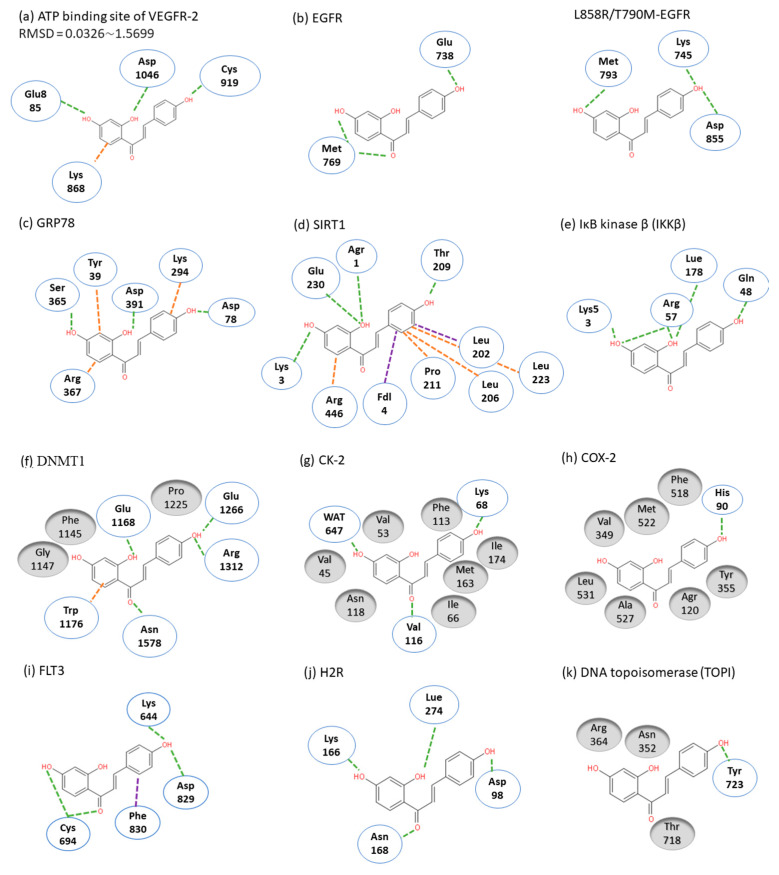
Molecular docking models. Interactions are represented in green (hydrogen bonding), orange (π–π stacking), purple (sigma-π) dash lines and gray (hydrophobic interaction: Van der Waals). (**a**) VEGFR-2; (**b**) EGFR; (**c**) GRP78; (**d**) SIRT1; (**e**) IKKβ; (**f**) DMNT1; (**g**) CK-2; (**h**) COX-2; (**i**) FLT3; (**j**) H2R; (**k**) TOPI.

ISL had been reported to exert diverse biological properties, but the specific molecular interaction that underlies these activities has not been fully unveiled. Based on molecular docking analysis, many studies have proposed that ISL has a direct interaction in different molecules (Figure 3), such as SIRT1 [38], VEGF2 receptor [39], GRP78 [40], FLT3 [41], EGFR [42], IKKβ [43], Toll-like receptors (TLRs) [44], CK-2 (IC_50_: 17.3 µM) [45], H2R [46], COX-2 [47], aromatase (Ki: 2.8 µM) [48,49], topoisomerase I [50] and DNMT1 [51]. These docking results imply that the binding pocket is composed of hydrophobic regions and is stabilized by a hydrogen bond with its neighboring carbonyl group. The hydrogen bond interactions and π–π stacking contribute to a tight interaction with the binding site. These docking results provide valuable information about the binding interactions of ISL and the active site, although more studies are required to approve them. Using a bioassay-guided purification method, suggested that isolated ISL acts as a xanthine oxidase inhibitor (IC_50_: 55.8 µM; Ki: 17.4 µM) to avoid transplantation rejection and ischemia reperfusion damage [52]. In brief, multiple docking candidates indicate that ISL exhibits multiple biological properties and serves as a potential lead compound for developing new therapy in cancer treatment.

### 2.5. ISL Biology Effects

**Figure 4:**
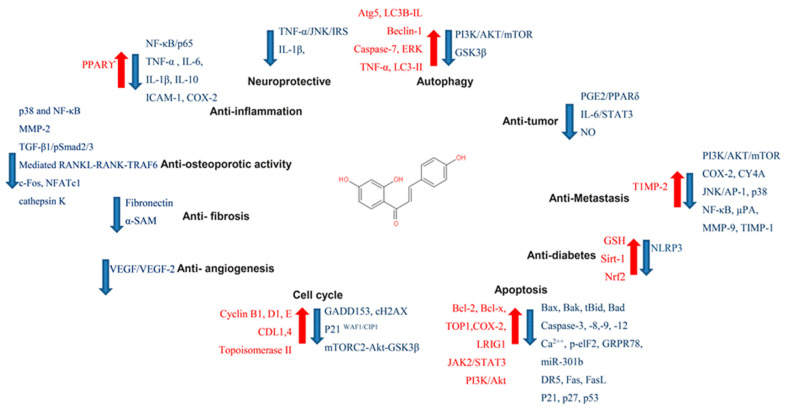
Pharmacological effect of ISL. The scheme presents the biological effects of ISL and molecular mechanisms of ISL against cancer via various signal pathways.

**Figure 5:**
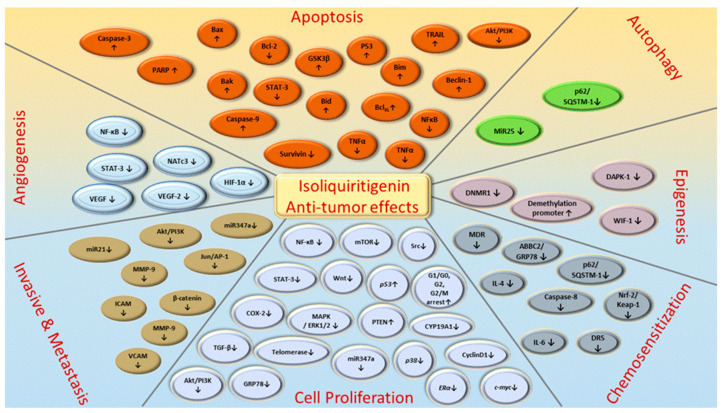
ISL-mediated regulation of molecular targets underlying anti-tumor effects, including tumor proliferation suppression, apoptosis induction, EMT/metastasis, epigenetic responses and sensitization to chemotherapy. Downward arrows (↓) represent downregulation while upward arrows (↑) represent upregulation. This figure was modified from [55].

In targeting cancers, ISL possesses various biologic activities, such as anti-inflammation, antioxidation, antiviral, antidiabetic, neuroprotective effect, chemopreventive, and antitumor growth properties (Figure 4 and Figure 5). A selective cytotoxicity effect of ISL has been reported (Table 2 and Table 3), and the effective dose in tumor cell lines shows very little cytotoxic effect on normal cells. Most studies have claimed that ISL significantly inhibits the viability of cancer cell but has little toxicity on normal cells. For example, Wu et al. (2017) compared the human endometrial stromal cells (T-HESCs; as a control) and human endometrial cancer cell lines (Ishikawa, HEC-1A, and RL95-2 cells). Their results indicated that ISL inhibits the growth of cancer cells at concentrations below 27 μM, but has little effect on normal cells [53]. Na et al. (2018) claimed that ISL shows little toxicity on normal hepatocyte cell lines (AML-12); only when applied in concentrations of over 100 μM is ISL harmful to normal hepatocytes [54]. Most studies have focused on the cytotoxicity between tumor and normal cells, and the effects of ISL on normal cells remain unknown. As Peng et al. (2015) mentioned, further research on the target organ toxicity or side effects of ISL is needed. The safety of ISL is always one of the most important concerns that must be evaluated.

## 3. ISL Anti-Tumor Effects

Many previous studies have provided evidence that ISL has anticarcinogenic activity in various types of cancers, including breast cancer, colon cancer, gastrointestinal cancer, lung cancer, ovarian cancer, leukemia, and melanoma. In Table 3 and Table 4, we summarize the research progress regarding the ISL’s antitumor activity in vitro and in vivo, respectively.

### 3.1. ISL’s Effects on Breast Cancer

From the WHO database (https://www.who.int/cancer), breast cancer is the most common cancer among women, impacting 2.1 million women each year. Breast cancer is still regarded as the second leading cause of cancer death in women. In the 2018 cancer statistics, it was estimated that 627,000 women died from breast cancer, which accounted for approximately 15% of all cancer deaths among women. Breast cancer can be triggered by multiple factors such as cancer stem cells (CSCs), the tumor microenvironment, genetic and epigenetic abnormalities, and so on. Most typical types of breast cancer are based on the expression of the ER-positive type of estrogen receptor (ER). Above two-thirds of breast cancers are termed hormone-dependent breast cancers, which rely on estrogen for tumor growth. Hormonal therapy or aromatase inhibitors are commonly applied in ER-positive breast cancer. Aromatase inhibitors may exert tumor-suppressing effects, preventing the conversion of androgen into estrogen. According to the function of aromatase inhibitors, previous studies have strongly suggested that ISL can act as an aromatase inhibitor [39,48,49,70] for a breast cancer remedy. However, another type of breast cancer, known as basal-like or triple-negative breast cancer (TNBC), does not respond to hormonal therapy. The advanced treatments in breast cancer include radiation, surgical exclusion, and the use of various chemotherapeutic drugs such as paclitaxel, doxorubicin, cisplatin, epirubicin, and 5-FU (5-fluorouracil). However, the incidence of drug resistance and serious side-effects associated with these treatment methods has greatly reduced their therapeutic potential. Therefore, alternative and safer chemotherapeutic strategies are needed.

Doxorubicin is one of the most effective agents for a wide spectrum of cancers, including breast cancer. The mechanism of doxorubicin is the inhibition of the DNA topoisomerase I & II and DNMT1, the same as ISL [50,51,141]. However, when treated with doxorubicin, patients suffer from serious cardiotoxicity and drug resistance. Lin et al. (2017) demonstrated that treatment with ISL alone or in combination with doxorubicin is highly effective in sensitizing doxorubicin-resistant cancer cells, resulting in the reduced survival of cancer cells [142]. Moreover, ISL not only inhibits cancer cell growth by inducing apoptosis and autophagy, but can also enhance chemosensitivity [40,66,67,69]. It has also been reported that doxorubicin triggers an epithelial-to-mesenchymal transition (EMT) in TNBC through mediating the PI3K/AKT pathway. Interestingly, ISL also can inhibit the PI3K/AKT pathway and thus suppresses EMT and increases the antiproliferative effect [40,66,67,69]. ISL or its derivatives show a greater influence by regulating the miR-374a/BAX axis, the -374a/PTEN/AKT axis, or the autophagy-mediated apoptosis (p62/caspase-8) pathway, especially in TNBC [33,67,69]. Recent studies have also demonstrated that ISL causes chemosensitization and induces autophagy following the degradation of the ABCG2 autophagy–lysosome pathway or the miR-25-mediating ULK1 (a kinase involved in autophagy) [40,56]. To further prevent the invasion in breast cancer, upregulating RECK (tumor suppressor gene) and downregulating miR-21 has been reported [65,68]. ISL has been suggested to be a supplement with chemotherapy or an alternative therapeutic agent for clinical trials against breast cancer, thereby warranting further investigation. The other first-line chemotherapies for breast cancer are epirubicin, 5-FU, and Taxol. Remarkably, previous studies have also shown that ISL can interact synergistically with these first-line chemotherapy drugs through mediating cell death (apoptosis) and autophagy and suppressing breast CSCs [40,56,69]. In a preclinical study, ISL was able to shed a novel light on reversing the epigenetic changes of Wnt inhibitory factor 1 (WIF-1), which induced the demethylation of WIF-1 promoter and subsequently prevented tumor initiation by inhibiting CSCs [51]. Based on the research described above, ISL greatly enhances the therapeutic efficacy of different chemotherapy drugs, overcomes drug resistance, and achieves sensitization to radiation (Table 5).

Even without combination treatment, ISL alone possesses anticancer activities in multistage carcinogenesis processes, including proliferation suppression, cell cycle arrest, angiogenesis inhibition, metastasis obstruction, apoptosis induction, autophagy induction, and metabolism (arachidonic acid and glucose metabolism). The administration of ISL alone to xenograft animals significantly inhibits lung metastasis in breast cancer and suppresses the expression of matrix metallopeptidase-9/7/2 (MMP-9/7/2), NF-κB, and cyclooxygenase-2 (COX-2) [57,63,64,66]. Concerning the inhibition of the tumorigenesis and metastasis of breast cancer, ISL can rectify the abnormal PI3K/AKT, NF-kB, and p38 signaling pathways in order to reduce the occurrence of metastasis through correcting the expression of MMP-2, MMP-7, MMP-9, VEGF, and HIF-1α [39,57,65,66,67]. Moreover, ISL hampers breast cancer growth and the neoangiogenesis accompanying suppressed VEGF/VEGFR-2 signaling, which prompts HIF-1α proteasome degradation or directly blocks VEGF-2 (Figure 3) [39]. ISL inhibited the multiple mRNA expression of phospholipase A2 (PLA2), cyclooxygenases-2 (COX-2), and cytochrome P450 (CYP) in an arachidonic acid (AA) metabolic network, as well as decreased the secretion of prostaglandin E2 (PGE2), 20-hydroxyeicosatetraenoic acid, and phosphorylation of PI3K. Meanwhile, in an in vivo test, ISL interferes with the AA metabolic enzyme to suppress the tumor growth of MDA-MB-231 human breast cancer xenografts in nude mice [66].

### 3.2. Effects on Colon Cancer

Colorectal cancer (CRC) is a common and lethal disease. In 2020, ~18,000 cases of colorectal cancer were diagnosed in people under 50—the equivalent of 49 new cases daily. Moreover, it is expected that 10 people die from CRC daily [150]. Generally, CRC develops in the colon or rectum, causing by both environmental and genetic factors such as old age and lifestyle. Some studies have demonstrated that CRC cells show increased proliferation, migration, and invasion in the presence of an acidic tumor microenvironment (TME), which further hinders chemotherapy [62,151]. In an acidic tumor microenvironment, fructose-bisphosphate aldolase A (ALDOA), pyruvate kinase muscle isozyme M2 (PKM2,) and lactate dehydrogenase A (LDHA) are overexpressed in colon cancer, resulting in high acidity of the intracellular environment. LDHA overexpression could engender hypoxia-inducible factor 1-alpha (HIF-1α) stability to enhance the generation of glycolysis [152,153]. To inhibit glycolysis and lactate generation in a tumor, ISL mediates HIF-1α stability and inhibits the AMPK and AKT/mTOR pathway. This phenomenon had been found in colon cell lines and mouse melanoma B16F10 cells [27,103]. More importantly, this downregulation of AA-metabolizing enzymes and the deactivating PI3K/AKT phenomena can also be observed in MDA-MB-231 human breast cancer xenografts in nude mice in vivo [66]. ISL not only affects the metabolic pathway, but it also inhibits tumor growth via prompting apoptosis and autophagy. In the study of Auyeung et al. (2010) [74], ISL inhibited tumor growth throughout the downregulation of the antiapoptotic proteins Bcl-2 and Bcl-x(L), arrested in G2. Moreover, ISL remarkably reduces PGE2 and nitric oxide (NO) production to induce apoptosis in mouse and human colon carcinoma cells [76]. Compared to the chemotherapy treatments in colon cancer, capecitabine, 5-FU, and gemcitabine act as antimetabolites, interfering with DNA synthesis. As mentioned above, ISL can abate the metabolism and possesses a DNA demethylation effect [71]. However, chemotherapy frequently results in a resistance issue. In a preclinical study, combinations of ISL with other chemotherapy drugs were tested [72,73,75,103]. Additionally, ISL has been identified as a potential multidrug-resistant (MDR) modulator candidate due to its ability to regulate the expression of the ABCB1, ABCC1, caspase 3, caspase 8, AhR, CYP1A1, and GSTP1 genes in colon-MDR cells [11]. Thus, a combined treatment in chemotherapy-resistant cells mediates the apoptosis/cell death of resistant cells. Furthermore, it was the first application to combine tumor necrosis factor-related apoptosis-inducing ligand (TRAIL) with ISL successfully to observe the chemopreventive effects of ISL. Its mechanism is dependent on the amount of death receptor 5 (DR5) protein among the TRAIL receptors. However, this means that the induction of apoptosis primarily relies on the TRAIL function [72]. In vitro, ISL can also mediate p53, EGRF-MAPK, and NAG-1 expression (Table 3) against colon cancer. Moreover, ISL downregulates ROS, NO-production, NF-κB activity, PGE-2, and COX-2 (see more details in Table 3) for tumor suppression.

### 3.3. Effect on Ovary Cancer

Ovary cancer, the most lethal of all gynecologic malignancies due to the limitation of early detection, presents in postmenopausal women with months of abdominal pain. The overall five-year relative survival rate of invasive epithelial ovarian cancer at diagnosis is approximately 40%. Worldwide, 2.2 million women have developed epithelial ovarian cancer every year [154]. Some prospective case–control studies have found that genetic mutation, endometriosis, human papillomavirus, perineal talc, and smoking are the risk factors that increase the incidence of ovarian cancer. Surgery and platinum-based cytotoxic chemotherapy are the standard of care for ovarian cancer therapy [154]. Ovarian cancer can reoccur and cause death due to the high metastatic and spread rates to the organs in the abdominal, brain, or lymph nodes outside of the abdomen. Therefore, controlling ovarian cancer metastasis is considered one of the most effective therapeutic strengths. ISL alone or in combination with other chemotherapeutic agents has been applied for the treatment of ovarian cancer. In an in vitro study, ISL treatment inhibited cell proliferation and induced cell apoptosis in ovarian carcinoma. The IC_50_ values of ISL on SKOV-3, OVCAR-5, and ES2 cells were 83.2, 55.5, and 40.1 μM, respectively (Table 3). ISL at 10 µM deterred ovarian carcinoma cells’ epithelial-to-mesenchymal transition (EMT), migration, and invasion through increasing the protein expression of E-cadherin and reducing the levels of ZEB1, vimentin, and TGF-β. ISL at 10 µM can suppress the intraperitoneal xenograft development of ovarian cancers [79]. Furthermore, ISL also induces ovarian cancer cell apoptosis through inducing oxidative stress, increasing endoplasmic reticulum stress, and leading to excessive intracellular ROS generation. This effect can be alleviated by co-treatment with Z-ATAD, a caspase-12 inhibitor [83].

### 3.4. Effect on Lung Cancer

Lung cancer, also known as lung carcinoma, is the leading cause of cancer-related deaths worldwide due to the detection at an advanced stage [155,156]. There were more than 230,000 new cases found in U.S. alone in 2018. There are two main types of lung cancer: non-small-cell lung cancer (NSCLC; approximately 80–85%) and small cell lung cancer (SCLC; approximately 10–15%). Long-term tobacco smoking is the predominant risk factor of lung cancer. Previous studies have implied that approximately 80–90% of all cases of lung cancer are caused by cigarette smoking or passive smoking. Other risk factors are chronic obstructive pulmonary disease (COPD), family history, gender (men), and exposure to radon, asbestos, or carcinogens. The mutation of the genes *EGFR, KROS, MET, LKB1, BRAF, PIK3CA, ALK, RET*, and *ROS1* is associated with the development of lung cancer [157]. EGFR inhibitors possess significant clinical benefit to NSCLC patients. ISL has been found to inhibit cell proliferation and cell cycle arrest in the A549 cell line, a human NSCLC cell line, through the activation of the p21CIP1/WAF pathway (IC_50_ = 18.5 or 27.14 µM). This result was comforted by other papers [90,91]. ISL (20 µM for 24 h) inhibits cancer cell migration and induces cell cycle arrest through the inhibition of the mTOR via PI3K/AKT pathway, which is one of the primary anti-apoptotic pathways activated by EGFR. Moreover, ISL downregulates the following protein levels: p21, Bax, Bcl-2, and p53, the most important cell cycle regulator in the A549 cell line. ISL has been found to inhibit E-cadherin P70, cyclin D1, N-cadherin, and vimentin, and thus suppress EMT [136,158]. Furthermore, an in vivo study provided similar results: Tumorigenesis was reduced in six-week-old athymic nude mice after IP injection with ISL (1 or 5 mg/kg, three times per week for two weeks). This phenomenon occurs, at least in part, through targeting with EGFR, thereby reducing the suppressed AKT and ERK1/2 signal pathways [42]. Inflammation has been demonstrated to play a major role in cancer development. Anti-inflammatory drugs have been considered as cancer therapeutic agents. ISL has been found to possess an anti-inflammatory effect, both in vivo and in vitro [86,136,137,138,139].

### 3.5. Effect on Leukemia

The anticancer activity of ISL on leukemia has also been evaluated. In in vitro studies, ISL at 50 µM significantly inhibited lymphocytic leukemia (LCL) cell proliferation after a 24 h administration. This effect occurs, at least in part, through the inhibition of p53 and cell cycle (estimated IC_50_ = 40~65 µM) [84]. Furthermore, ISL also abolishes cell proliferation and induces cell differentiation by the upregulation of antioxidative activity in HL-60 cells (estimated IC_50_ = approximately 40.42 µM) [85,94,95]. Similarly, ISL induces cell cycle arrest in the G2/M phase in the human T cell leukemia Jurkat and CCRF-CEM cell line (IC_50_ = 18.38 μM) [96,97]. In addition, ISL also inhibits DNCB-induced pro-inflammatory cytokines secretion, as well as p38-ERK signaling, in human monocyte model THP-1 cells [98]. ISL also decreases inflammatory cytokine secretion through the inhibition of the TRIF-dependent pathway in RAW264.7 cell line [92,93]. In in vivo studies, 30-day oral administration of ISL significantly inhibits MV4-11 flank tumor growth and prolongs survival via decreasing cell proliferation and inducing apoptosis [41]. Oral administration of ISL in experimental AD-like lesion model mice significantly suppresses DNCB-induced IgE and Th2 cytokine upregulation [98]. ISL possesses an immune-suppressive effect directly on human T cells via covalent binding of IKKβ Cys46 without significant toxicity [43]. A preclinical study on the T-ALL cell line showed that ISL inhibits the survival of doxorubicin or methotrexate-resistant cell lines. Therefore, ISL may be a valuable adjunct for cancer therapy to treat otherwise drug-resistant tumors [96]. The anti-inflammatory effect of ISL also implies that it can be applied in allergic asthma patients [147].

### 3.6. Effect on Melanoma

The antitumorigenic effects of ISL on melanoma have also been evaluated extensively. Xiang et al. found that ISL inhibits cell proliferation and induces cell apoptosis through stimulating the expression of C-PARP, Bax, and cleaved-caspase-3 [99]. It also induces B16F0 melanoma cell differentiation. Three pathways, i.e., the glutathione metabolism, glycolysis/gluconeogenesis, and pentose phosphate pathways, are the most important pathways perturbed by ISL [100]. Moreover, ISL can activate the mTORC2-AKT-GSK3β signaling pathway, thereby inducing cell cycle arrest, reprogramming A375 melanoma cells (estimated IC_50_ = ~48 µM) [101]. ISL can decrease the expression of mitochondrial protein mitoNEET, thereby decreasing mitochondrial membrane potential, altering ROS content, and subsequently inducing cell apoptosis in A375 cells (estimated IC_50_ = ~73 µM) [102]. In contrast, Wang et al. found that ISL can stimulate ROS, leading to oxidative stress, thereby inducing B16F10 cell apoptosis (estimated IC_50_ = ~35 and 22 µg/mL) [103]. Similarly, ISL increases ROS accumulation and facilitates melanogenesis, thereby stimulating B16F10 cell differentiation [104]. ISL can inhibit the growth of human as well as murine myeloma cell lines via inhibiting IL-6 signaling (*p*-ERK, *p*-STAT3, etc.), inducing cell apoptosis and cell cycle arrest [105]. In SCID mice bearing U266, BABL/c bearing MPC-11, or murine myeloma xenograft models, the antitumor activity of ISL has also be found by ISL alone or in combination with Adriamycin via blocking IL-6 signaling [105]. ISL also induces cell apoptosis in B16 melanoma mouse melanoma via the inhibition of glucose transmembrane transport [107]. Moreover, ISL exerts antimelanogenic effects through activating the phosphorylation of ERK and inhibiting tyrosinase activity in SK-MEL-2 and HaCaT cells [106].

### 3.7. Effect on Hepatoma

Hepatoma, also known as hepatocellular carcinoma (HCC), is the most common primary malignant tumor of the liver in adults. Hepatoma, which is mainly caused by cirrhosis, is the second leading cause of cancer-related death worldwide. Traditional herbal medicines, including licorice, have been widely used for HCC prevention and treatment. ISL, the compound purified from licorice, has been used in hepatoma treatment recently. As depicted in Table 3, ISL exhibits toxic effects on Hep3B hepatoma cells by inducing cell cycle arrest at the G1/S checkpoints, suppressing migration and metastasis and the PI3K/AKT signal pathway (IC_50_ = 42.84 ± 2.01 μM). Upon ISL treatment, the protein expression and kinase activity of the cell cycle regulators are altered in hepatoma cell lines [108]. Furthermore, ISL induces apoptosis via the MAPK/STAT3/NF-κB/IkB signaling pathway, ROS accumulation, and the p53-dependent pathway and reduces cell cycle-associated protein expression in HepG2 and Hep3B cells [109,110,111]. The effects of ISL on the hepatocellular carcinoma cell line Hepa 1c1c7 have been investigated, finding that cell growth decreases and apoptosis is induced in both Hep G2 and PLC/PRF/5 (IC_50_ = 36.3 μM) [112]. ISL has been shown to inhibit liver cancer cells (SK-Hep-1) proliferation (IC_50_ = 19.08 μM) [113]. ISL also inhibits DNA cleavage reaction via inhibiting TOP I activity in the SNU475 cell line [50]. In a xenograft model in female BALB/c- mice bearing Hep3B cells, when subjected to IP ISL (50 mg/kg/day for three weeks) administration, they showed decreased tumorigenesis and metastasis of HCC due to a reduction in the expression of cyclin D1 and the suppression of the PI3K/AKT pathway [108]. Similarity, a single IP administration of ISL (10 mg/kg) increases radiosensitization via the inhibition of the Nrf2/Keap1 pathway in four-week-old male athymic BALB/c (nude) mice bearing HepG2 [140].

### 3.8. Effect on Prostate Cancer

Prostate cancer is the most common noncutaneous cancer among men. It is also the second-leading cause of cancer deaths for men in the U.S. Alternative therapies are becoming increasingly popular among patients with prostate cancer. The realization that ISL has a role to play in the chemoprevention of prostate cancer has led to a number of cell line-based investigations aimed at understanding the mechanism of ISL (Table 2). ISL induces cell apoptosis in prostate cancer cells through G2/M cell cycle arrest with concomitant downregulation of cyclin B1, CDK1 (p-Thr14, p-Tyr15, and p-Thr161) (after 48 h of treatment, the IC_50_ of ISL on PC-3 and 22RV1 is 19.6 and 36.6 μM, respectively) [114]. Zhang et al. observed that ISL (IC_50_ = 87.0 μM) inhibits the anticancerous effects on C4-2, LNCaP prostate cancer cells, by the reduction of the Psi(m) that triggers apoptosis and the inhibition of the proliferation via the ERK/p38MAPK pathway [59]. The effects of ISL on prostate cancer cell line DU145 have been investigated, finding that cell cycle arrest in the G2M phase decreases CDC25C and increases p-CDC2 (Tyr15), cyclin B1, and p27^KIP1^ [115]. The anti-invasion and antimetastasis of ISL on the DU145 prostate cancer cell line have been investigated, with the findings suggesting that this mechanism could be achieved through the inhibition of JNK/AP-1 signaling and the downregulation of the reduction of µPA, MPP-9, and AP-1. Moreover, the expression of some proteins, including VEGF, integrin-α2, and ICAM, and VCAM, has also been shown to be reduced [116]. Furthermore, the inhibition of the PI3K/AKT and HRG-β-induced ErbB3 signaling pathways has also been found in the DU145 cell line [117]. ISL can induce prostate cancer cell apoptosis via increasing Fas ligand (FasL), Fas, cleaved casapse-8, tBid, cytochrome c, and Smac/Diablo (ISL shows an estimated IC_50_ of 13.74, 5.67, and 5.01 µM in the MAT-LyLu cell line treated for 24, 48, and 72 h, respectively; in contrast, it shows an estimated IC_50_ of 56.87, 31.49, and 17.60 µM in the MAT-LyLu cell line treated for 24, 48, 72 h, respectively) [118]. The effects of ISL on prostate cancer cell apoptosis have been investigated, with researchers finding that ISL induces DU145 and LNCaP cell cycle arrest in the G2/M stage through increasing the expression of GADD153 mRNA (estimated IC_50_ of ISL on Du145 and LNCaP is 10.56 and 10.78 µM, respectively) [119]. An antitumor effect of ISL against prostate cancer in an animal model has been reported. In prostate-tumor-bearing animals, i.e., male BALB/c nude mice bearing PC-3, they were treated with 25 or 50 mg/kg/day ISL for 28 days; a decrease in cyclin B1–CDK1 and G2/M arrest and apoptosis was detected [114].

### 3.9. Effect on Cervical Cancer

Cervical cancer is the fourth most common cancer in women worldwide and causes more than one quarter of a million deaths per year. Several studies have also focused on the anti-cervical cancer effects of ISL. ISL induces intrinsic apoptosis and S-phase arrest in Ca Ski, SiHa, HeLa, and C-33A cells. ISL inhibits proliferation and induces cell cycle arrest in the G2/M phase, which may be attributed to the decreased expression of Bcl2 and the increased expression of caspase-related proteins and cell cycle checkpoints, including p53, p21, Bax cyclin B, cyclin A, cdc2, and cdc25C (estimated IC_50_ = 39.09 μM in Ca Ski; 53.76 μM in SiHa; 9.8 or 58.10 μM in HeLa; 32.83 μM in C-33A cells) [120,122]. ISL also induces apoptosis in cancer cells through increasing ROS generation, *p*-eIF2α and GRP78 expression, and caspase-12 activation in HeLa cells (estimated IC_50_ = approximately 21.24 μM) [121]. ROS production is important for the anticancer activity of ISL in HeLa cells. This was proven by Yuan et al., showing that the apoptotic rate is increased after co-treatment of ISL and pro-oxidant, l-buthionine-(S,R)-sulfoximine (BSO). On the contrary, the apoptosis rate is inhibited by co-treatment with free radical scavenger N-acetyl-cysteine (NAC) [144]. In an in vivo study, in KM mice bearing U14, when administered in combination with cyclophosphamide, ISL enhanced the antitumor activity and decreased the micronucleus formation of DNA strand breaks [145].

### 3.10. Effect on Other Cancers

Studies have shown that ISL also induces call apoptosis and/or autophagy in other cancers, including gastric cancer, uterine leiomyoma, osteosarcoma, glioma, bladder cancer, and oral squamous cell carcinomas (OSCC). In gastric cancer, ISL causes cell apoptosis and autophagy in MKN28 cells by suppressing the PI3K/AKT/mTOR pathway and by increasing Beclin-1 (IC_50_ = 20.84 µM) [123]. ISL (5 µM) negatively affects H2R-mediated c-Fos/c-Jun protein expression, acting as an effective H2R antagonist in the MKN-45 cell line [46,124]. ISL (0.11 g/L for 24 h) induces MGC-803 cell apoptosis via the calcium- and Delta psi(m)-dependent pathways [125]. In uterine sarcoma, the combined treatment of human uterine sarcoma cell line MES-SA cells with ISL and doxorubicin significantly enhances chemosensitivity via inducing apoptosis and autophagy by inhibiting the mTOR pathway [142]. In uterine leiomyoma, ISL induces cell cycle arrest in the subG1 and G2/M phases by increasing p21Cip1/Waf and reducing Bcl-2, cdk 2/4, and E2F, thereby suppressing the proliferation of primary uterine leiomyoma cells. ISL also induces cell apoptosis through the elevation of FAS ligand and caspase-3 (estimated IC_50_ = 39.33 µM) [126]. In contrast, a low cytotoxicity of ISL has been found in normal myometrium cells (estimated IC_50_ = 698.8 µM). In osteosarcoma, ISL has also been shown to cause DNA damage and can initiate apoptosis through increasing Bax and caspase 3 and cell cycle regulators, including p53, p21, and p27, in U2OS and Saos‑2 cells (main dosage of 20 and 30 μM, respectively) [127,128]. In glioma, ISL induces cell apoptosis by the elevation of caspase 3 and the inhibition of TOP I in glioma U87 cells (IC_50_ = 6.3 µM) [130]. In SK-N-BE(2) an IMR-32 neuroblastoma cell line, ISL (>5 µM) has been found to increase the ROS level, thereby inducing cell death. The combined treatment of SK-N-B-E(2) cells with ISL and the anticancer agent cisplatin significantly reduces cell viability compared to cisplatin alone [129]. ISL inhibits cancer growth and induces apoptosis and autophagy in PC12 cells by dose-dependently downregulating Bcl-2 and Bcl-x and by stimulating caspase-9, caspase-3, caspase-7, Bax, Bim, cytochrome c, Beclin-1, and LC3 expression (IC_50_ = 17.8 ± 1.8 μM) [131]. In bladder cancer, pretreatment with ISL for 24 h enhances cisplatin-induced cell death, ROS production, the upregulation of Bax, Bim, Apaf-1, caspase-9, and caspase-3 levels, and the downregulation of Bcl-2 levels in the T24 cell line. In contrast, it attenuates cisplatin-induced proximal tubular cell (LLC-PK1) injury by upregulation of HO-1 levels [132,148]. In LLC-PK1 porcine kidney cancer cells, pretreatment with ISL induces ER stress and produces hormesis to protect against cisplatin-induced nephrotoxicity [149]. Oral cancer is defined as any malignant cell growth in the oral cavity. OSCC comprises more than 90% of oral cancer cases, and is the third most prevalent malignancy in developing countries. In our previous study, we found that ISL induces OSCC cell cycle G2/M phase arrest, apoptosis, and DNA damage through the inhibition of ATM signaling. A low dose of ISL (6.25 μM) inhibits OSCC malignancy in vitro. ISL (5 mg/kg) also reduces the tumor size in vivo [159]. ISL downregulates GRP78 levels, thereby suppressing oncogenicity both in vitro and in vivo. The combination of ISL and cisplatin significantly represses the invasion and colony formation abilities of OSCC cells by downregulating the expression of CSC markers and ABC transporters [60].

## 4. Conclusions

**Figure 6:**
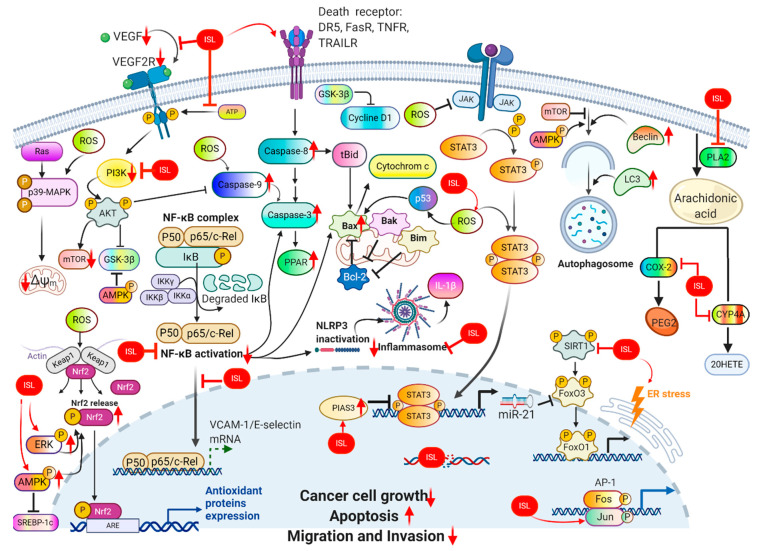
ISL exhibits significant anticancer activity through various mechanisms, such as proliferation suppression, apoptosis induction, and/or autophagy, and inhibits migration and invasion in various cancer cells.

ISL exhibits significant anticancer activity through various mechanisms, such as proliferation suppression, apoptosis induction, and/or autophagy, and inhibits migration and invasion in various cancer cells (Figure 6). Licorice Kampo and ISL are not only potential candidates for adjuvant chemotherapy, but also possess anticancer properties. However, clinical trials using ISL against cancer have not been initiated. Undoubtedly, both in vitro and in vivo studies have demonstrated the potential of ISL for the prevention and treatment of different types of cancers (Table 3 and Table 4). With encouraging outcomes in preclinical studies, many studies have strongly emphasized that ISL can increase the chemosensitivity of different kinds of chemotherapies (Table 5). More, the application of ISL in the form of nanoformulations as a novel strategy in order to improve its efficacy (Table 1) is under ongoing development. Overall, the various research works have not only highlighted the significant anticancer activity of ISL in vitro and in vivo, but have also proposed various molecular-based interactions (Figure 3) underlying anticancer mechanisms. Overall, ISL is a promising candidate for a natural product with therapeutic effects and with the ability to alleviate the adverse side-effects in anticancer therapeutics in spite of the suppressive effects of ISL on different cancers in clinical trials being limited still.

## Figures and Tables

**Table 1:** Nano-formulation of ISL.

Formulation	Material	Particle Size (nm)	Model	Conclusion	Ref
Nanosuspension	Hydroxypropyl cellulose-SSL Polyvinylpyrroli-done-K30	238.1 ± 4.9 354.1 ± 9.1	In vitro: A549	HPC SSL‑ISL‑NS and PVP K30-ISL‑NS both improve the solubility and cytotoxic activity of ISL (IC_50_: ~0.08 µM).	[20]
Nanoencapsulation	Mesoporous silica nanoparticles	~200	In vitro: mouse primary bone marrow-derived macrophages (BMMs) In vivo: lipopolysaccharide (LPS)-mediated calvarial bone erosion model (received 50 mg/kg MSNs-ISL; once every 2 days via subcutaneous injection) Experiment period: 7 days	MSNs-ISL as an effective natural product-based bone-bioresponsive nanoencapsulation system prevents osteoclast-mediated bone loss (In vitro effective dose: 16~64 µg/mL).	[21]
Lipid–polymer hybrid	ISL-iRGD nanoparticles	~130 138.97 ± 2.44	In vitro: MCF-7, MDA-MB231, 4T1 In vivo: 4T1-bearing nude mouse (received 35 µg/kg once every 2 days via IV injection) Experiment period: 20 days	RGD modified lipid–polymer hybrid NPs improve ISL in anti-breast cancer efficacy (Effective dose: >12 µM).	[22]
	LMWH-ISL-SLN	217.53 ± 4.86	In vitro: HepG2 In vivo: Kunming mice (6 female and 6 male; 50 mg/kg via IV injection daily) Experiment period: 14 days	Pharmacokinetics of LMWH-ISL-SLN demonstrated its safety and better bio-distribution after intravenous administration (In vitro IC_50_: ~7.45 µg/mL).	[23]
Micro-emulsion	Self-microemulsifying drug delivery system (SEMDDS)	44.78 ± 0.35	In vivo: SD rat (oral administration: a single dose: 200 mg/kg) Experiment period: 24 h	ISL-SMEDDS can enhance the solubility and oral bioavailability of ISL.	[17]
		20.63 ± 1.95	In vivo: SD rat (oral administration: twice a day; 20 mg/kg) Experiment period: 63 days		[29]
Nanostructured lipid carrier (ISL-NLC)	Monostearate and lecithin	160.73 ± 6.08	In vivo: Kunming mice bearing H22 and S180 tumor (intraperitoneal injection daily) Experiment period: 12 days	ISL-NLC nanoparticles with high envelopment efficiency with initial burst release, exhibiting superior in vivo antitumor effect and biodistribution.	[24]
	MS and Miglyol 812	160.73 ± 6.08	In vivo: SD rat (oral administration: a single dose: 20 mg/kg) Experiment period: 36 h	NLC are valuable as an oral delivery carrier to enhance the absorption of a poorly water-soluble drug, ISL.	[19]
	Ceramide, cholesterol, caprylic/capric triglyceride	150.2–251.7	In vitro: Franz diffusion cell In vivo: ICR mice	NCL improved the skin permeation of ISL (permeability: 8.48~10.12 μg/cm^3^).	[25]
TPGS-modified proliposomes	D-α-tocopheryl polyethylene glycol 1000 succinate (TPGS), proliposomes	23.8 ± 0.9	In vivo: Swiss-ICR mice oral administration Experiment period: 24 h	ISL-TPGS-PLP had small particle size, high encapsulation efficiency and drug loading capacity, and possessed good storage stability.	[18]
Polymeric micelles	ISL-loaded F127/P123 polymeric micelles (ISL-FPM)	20.12 ± 0.72	In vivo: SD rat, (oral administration: a single dose 200 mg/kg) Experiment period: 24 h	ISL-FPM act as a promising approach to improve solubility as well as enhance bioavailability and antioxidant activity of ISL.	[26]
Liposome	Phospholipid and cholesterol	233.1	In vitro: HeLa and SiHa	ISL liposome can significantly inhibit the proliferation of human cervical cancer cells in vitro.	[30]
Nanoliposome	Sodium cholate, cholesterol and IPM were melted with a ratio of 5:1:4 (w/w/w)	82.3 ± 35.6	In vitro: HCT116 and HT29	ISL involved in the glucose metabolism in colon cancer.	[27]
Hydrogel systems	HA-HEC hydrogels	N.A.	In vitro: skin permeation study Franz diffusion cells	HA-HEC hydrogel showing the stable viscoelastic be haviour and the optimal adhesiveness has potential to enhance skin permeation of IS (permeability: 20 μg/cm^3^).	[28]

**Table 2:** ISL influenced on normal cell lines.

Type	Cell Line	Result	Ref
Breast	MCF-10A (0~50 µM) (24 h)	ISL had no significant influence on MCF-10A as human normal tissues.	[40]
	MCF-10A (0~100 µM) (24 h)	ISL had limited inhibitory effects on the proliferation in normal cell and did not show the chemosensitization effect with epirubicin.	[56]
	H184B5F5/M10 (0.1~10 µM) (6~48 h)	ISL did not influence the normal cell viability at the at 0.1~10 µM.	[57]
Lung	HELF (24~72 h)	Both pure drug of ISL and nanosuspension showed low toxicity to normal cells.	[20]
Hepatocyte	AML-12 (0~200 µM) (24 h)	5~50 μM of ISL increased cell proliferation, strong cytotoxicity was observed over 100 μM.	[54]
Uterus Endometrium	T-HESCs (5~100 µM) (24~48 h)	The viability of T-HESCs showed significant changes when ISL concentration over 75 μM was applied.	[53]
Gastric	GES-1 (20 µM) (48 h)	ISL exhibited a negligible effect on cell growth and cell viability exceeded 70%.	[32]
Endothelia	HUVEC	Over 10 µM of ISL is nontoxic with inhibiting the VCAM-1 and E-selectin.	[58]
Small intestine	IEC-6 (10~100 µM) (24 h)	No effect was observed in IEC-6 cells.	[59]
Oral	SG cell (25~400 μM) (24 h)	The half maximal effective dose (IC_50_) of ISL is 386.3 ± 29.7 μM.	[60]
Brain	H22	ISL had the potential to against glutamate-induced neuronal cell death (neuroprotective effect)	[36]

**Table 3:** Different pathways of various cancers regulated by ISL.

Type of Cancer	Cell	Testing Range/IC_50_	Signaling Pathways Effect of ISL (In Vitro)	Ref
Breast cancer	MCF-7	Testing conc: 10 nM~10 µM (5 days; 10 nM is sufficient)	⇧Presenilin2 (pS2) mRNA level ⇩Proliferation ⇩Estrogen receptor (ERα)	[61]
	MCF-7 MDA-MB-231	Effective conc: 25 µM and 50 µM (24 h)	⇧WIF1 ⇩DNMT1 ⇩β-catenin (⇩Metastasis) ⇩Wnt ⇩G0/G1 (Cell cycle arrested) ⇩Cyclin D1 (⇧Apoptosis) ⇩Survivin ⇩c-myc ⇩Oct-4	[51]
	MCF-7 MDA-MB-231 HUVEC	Testing conc.: 0, 20, 40, 60, 80, 100 µM	⇧HIF-1α proteasome degradation ⇩VEGF expression ⇩Cancer growth via VEGF/VEGFR-2 ⇩Neoangiogenesis via VEGF/VEGFR-2	[62]
		Tumor cell line: MCF-7 IC_50 estimated_ = ~33.39 µM MDA-MB-231 IC_50 estimated_ = ~35.64 µM (48 h)		
		HUVEC IC_50 estimated_ = ~75.48 µM		
	PMA-induced COX-2 in MCF-10A	Effective conc: 0.1 µM and 10 µM (24 h; 1 µM is sufficient.)	⇩COX-2 expression modulated ERK-1/2 signaling	[63]
	BT549 MDA-MB-231	Effective conc.: 10, 20, 40 µM (12 h)	⇧Cleaved caspase-3 & 9 (⇧Apoptosis) ⇩COX-2 (⇩Metastasis) ⇩CYP 4A, ⇩PGE_2,_ ⇩PLA2	[64]
	MDA-MB-231 Hs-578T	Effective conc.: ~20 µM	⇧RECK ⇩miR21 and ⇩MMP-9 (⇩Invasive)	[65]
Breast cancer	MCF-7 MDA-MB-231	Testing conc.: 0, 5, 10, 20 µM	⇩mRNA level of phospholipase A2 (PLA2), cyclooxygenases-2 (COX-2) and cytochrome P450 (CYP) 4A ⇩Cancer growth (⇩Arachidonic acid metabolism) ⇧Apoptosis ⇩PI3K/AKT pathway	[66]
		Tumor cell line: MCF-7 IC_50_ = 10.08 µM MDA-MB-231 IC_50_ = 5.5 µM (48 h)		
	MCF-7 MDA-MB-231	Testing conc.: 0, 6.25, 12.5, 25, 50, 100 µM	⇧PTEN (⇧Apoptosis) ⇧Bax (⇧Apoptosis) ⇧Caspase 9 ⇧MMP-7 (⇩Lung metastasis) ⇩miR374a (⇩Metastasis and ⇩proliferation) ⇩Bcl-2 ⇩*p*-GSK3β, AKT ⇩β-catenin (⇩Migration and ⇩invasion)	[67]
		Tumor cell line: MCF-7 IC_50_: 32.66 µM MDA-MB-231 IC_50_: 22.36 µM (24 h)		
	MDA-MB-231 Hs-578T	Effective conc.: 10 µM and 20 µM	⇧PIAS3 ⇩miR21 and ⇩STAT3 (⇩Invasion)	[68]
	MCF-7 MDA-MB-231 BT549 MCF-10	Testing conc.: 1, 5, 10 and 25 µM	⇧Proteasome degradation ⇧β-catenin degradation ⇧Apoptosis via ⇩ miR-374a ⇧Chemosensitivity ⇩β-catenin /ABCG2/ GRP78 (⇩Proliferation) ⇩GSK-3β phosphorylation via AKT pathway (⇧Chemosensitization) ⇩CD44^+^CD24^−^, Survivin, Oct-4, ⇩Cyclin D1	[40]
		Tumor cell lines: MCF-7 IC_50 estimated_: ~33.0 µM MDA-MB-231 IC_50 estimated_: ~21.2 µM BT549 IC_50 estimated_: ~18.1 µM (24 h)		
		Normal cell line: MCF- 10A IC_50 estimated_: ~80.51 µM (24 h)		
Breast cancer	MCF-7 MDA-MB-231 H184B5F5/M10	Effective conc: 25 µM and 50 µM (48 h) Tumor cell lines: MCF-7 MDA-MB-231	⇩VEGF (⇩Anti-angiogenesis) ⇩HIF-1α (⇩Proliferation) ⇩MMP-9 (⇩Migration) ⇩PI3K ⇩NF-kB ⇩p38	[57]
		Normal cell line: H184B5F5/M10 (ISL did not influence the viability)		
	MCF-7 MCF-7/ADR MCF-10A	Tumor cell lines: MCF-7 IC_50 estimation_: ~59.39 µM MCF-7/ADR IC_50 estimation_: ~38.86 µM (24 h)	⇧ULK1 (⇧Autophagy) ⇧LC3-II (⇧Chemosensitization) ⇩miR-25(⇧Autophagy) ⇩ABCG2	[56]
		Normal cell line: MCF-10A ISL (at 100 µM) had limited inhibitory effects on the proliferation		
	MDA-MB-231	Testing conc.: 0, 10, 25, 50 µM MDA-MB-231 IC_50 estimated_: ~24.23 µM (48 h)	⇧Bax ⇧Caspase-3 and ⇧PARP ⇧ p62, ⇧Beclin1, and ⇧LC3 (⇧Autophagy) ⇧Caspase-8 (⇧Autophagy and ⇧apoptosis) ⇩Cyclin D1 (⇩Proliferation) ⇩Bcl-2 G1 arrest	[69]
	MCF-7aro	Testing conc.: 0, 0.625, 1.25, 2.5, 5, 10 µM MCF-7aro IC_50_: 2.5 µM (24 h)	⇩mRNA level of aromatase ⇩CYP19 promoters I.4, I.3 and II activity	[48,70]
Colon cancer	HT29	HT29 ED_50_: 11.1 µg/mL (42.32 µM)	DNA demethylating effect	[71]
	HT29	Testing conc.: 0, 5,10, 20, 30, 40, 50 µM 40 µM was applied; (24 h)	⇧DR5(⇧Apoptosis) ⇩PI3K/AKT pathway	[72]
	HCT116 HT29 SW480	Testing conc.: 0,10, 20, 30, 40 µM HCT116 IC_50 estimated_ = ~42.41 µM Working conc.: 30 or 40 µM; (24 h)	⇧Apoptosis ⇧p62/SQSTM1 (⇧Autopage cell death) ⇧PARP cleavage ⇩Caspase-8 activation (⇧Apoptosis)	[73]
	HCT116	Testing Conc.: 0, 2.5,5, 10, 20, 40, 80, 160 µM HCT116 IC_50 estimated_: ~78.78 µM (48 h) HCT116 IC_50 estimated_: ~53.97 µM (72 h) HCT116 IC_50 estimated_: ~44.8 µM (96 h)	⇧NAG-1 expression mediated EGR-1, p53, ATF-3, Sp1 and PPARγ ⇧Apoptosis (Caspase dependent pathway) ⇩Bcl-2 and Bcl-x_L_ G2 phase cycle arrested	[74]
	CT26	Testing Conc.: 0, 10, 20, 40, 60, 80 µM CT26 IC_50 estimated_ = ~54.48 µM	⇧Serum nitric oxide, ⇧Lipid peroxidation levels and ⇧GSH levels ⇩ ROS ⇩Proliferation ⇩COX-2 (⇧Apoptosis)	[75]
	Colon26 RCN9 CoLo-320DM	Testing Conc.: 0, 5, 25, 100 µM (24, 48 h) Colon26 IC_50 estimated_ = ~17.55 µM (24 h) Colon26 IC_50 estimated_ = ~12.59 µM (48 h) RCN9 IC_50 estimated_ = ~41.73 µM (24 h) RCN9 IC_50 estimated_ = ~18.21 µM (48 h) CoLo-320DM IC_50 estimated_ = ~23.10 µM (24 h) CoLo-320DM IC_50 estimated_ = ~10.82 µM (48 h)	⇧Apoptosis ⇩PGE_2_ depends on ⇩COX-2 expression ⇩NO via (⇩iNOS)	[76]
Colon cancer	HCT116	Applied 20 µM (48 h)	⇧Bax and ⇧cleaved caspase-3 (⇧Apoptosis) ⇩PI3K/AKT signaling pathway ⇩Cancer proliferation, ⇩Invasion and ⇩migration ⇩Bcl-2, *p*-AKT, *p*-mTOR, CyclinD1	[77]
	Caco-2/TC-7	Caco-2/TC-7 EC_50_: 42 μM	⇧ HBD3 (human β-defensin-3) ⇧EGFR-MAPK pathway	[78]
Ovary cancer	SKOV3 OVCAR5 ES2	Testing conc.: 2, 4, 8, 16, 32, 64, and 100 µM SKOV3 IC_50_: 83.2 µM (72 h) OVCAR5 IC_50_: 55.5 µM (72 h) ES2 IC_50_: 40.1 µM (72 h) Effective Conc.: 10 µM	⇧E-cadherin ⇩ZEB1 mRNA ⇩Vimentin and ⇩N-cadherin (⇩EMT) ⇩TGF-*β*	[79]
	SKOV3 OVCAR5	Testing conc.: 0, 1, 5, 10, 20, 25, 50, 75, and 100 µM OVCAR5 IC_50_: 11 µM (48 h) ES2 IC_50_: 25 µM (48 h)	⇧Cleaved PARP, ⇧cleaved caspase-3, ⇧ Bax/Bcl-2 ratio, ⇧LC3B-II, and ⇧Beclin-1 ⇧CDK2 G2/M phase arrest ⇩Cyclin B1	[80]
	Antral follicle culture (female CD-1 mic)	Testing conc.: 0.6, 6, 36, and 100 μM	⇧STAR ⇩mRNA levels of cytochrome P450 steroid 17 α-hydroxylase 1 (⇩CYP17A1), cytochrome P450 aromatase (⇩CYP19A1)	[81]
	SKOV3 OVCAR3	Testing conc.: 5~80 μM 30 μM applied	⇧GSK3β ⇩*p*-AKT and *p*-mTOR ⇩P70/S6K, Cyclin D1 ⇩Wnt3a, ⇩p-ERK, ⇩PI3K/AKT/mTOR	[82]
	SKOV3	N.A.	⇧ER stress,⇧ *p*-eIF2α, GADD153/CHOP, GRP78, XBP1 expression, and cleavage of ATF6α (⇧Apoptosis and ⇧autophagy)	[80,83]
Lung cancer	H1299 H1975 A549	H1299 IC_50 estimated_: ~36.78~46.08 µM H1975 IC_50_: 48.14 µM A549 IC_50_: 75.08 µM (48 h)	⇩Src kinase activity (⇩Proliferation and ⇩migration)	[9]
	A549	A549: applied 20 µM (24 h)	⇧Bax and ⇧caspase-3 ⇧E-cadherin ⇩Bcl-2 ⇩mTOR (⇩PI3K/AKT pathway) ⇩P70, ⇩Cyclin D1, ⇩*N*-cadherin and ⇩vimentin	[84,85]
	RAW 264.7	Testing conc.: 5, 10, 20 µM for (Pretreated with 10mM of t-BHP for 18 h) RAW 264.7 (treated with t-BHP) EC_50_ = 10 µM (18 h)	⇧AMPK/Nrf2 signaling ⇧ Nrf2 and its target enzymes (e.g., ⇧HO-1, ⇧GCLM, ⇧GCLC, and⇧ NQO1) ⇩iNOS and ⇩COX-2 ⇩TNF-α, ⇩IL-1β, and ⇩IL-6 ⇩NLRP3 in a Nrf2-dependent pathway ⇩NF-κB (p65) via Nrf2-independent pathway	[86]
	Calu-3	Calu-3 cells were infected with PR8/H1N1 virus; [EC_50_] = 24.7 μM	⇧PPARγ (⇩Influenza virus infection) ⇧TNF-α, ⇧IL-1β, and ⇧IFN-β	[87]
	H1650 H1975 A549	H1650 IC_50 estimated_: ~26.88 µM (24 h) H1975 IC_50 estimated_: ~8.92 µM (24 h) A549 IC_50 estimated_: ~46.7 µM (24 h)	⇧Bim (⇧Apoptosis) ⇩Bcl-2, ⇩ *p*-AKT, and ⇩*p*-ERK1/2	[42]
	A549	A549 IC_50_: 0.05 mg/mL (~191.21 µM ~117 µM)	⇧p53, ⇧p21 and ⇧Bax Arrest at G2/M phase ⇩PCNA, ⇩ MDM2, ⇩*p*-GSK-3β, ⇩*p*-AKT, ⇩*p*-c-Raf, ⇩*p*-PTEN, ⇩caspase-3, ⇩pro-caspase-8, ⇩pro-caspase-9, ⇩PARP, and ⇩Bcl-2	[88]
Lung cancer	guinea-pig tracheal smooth muscle	N.A.	⇧cGMP/PKG (⇧BKCa channels opened) ⇩PDEs (⇩[Ca^2+^]_i_ led tracheal relaxation)	[89]
	A549	A549 IC_50_: 27.14 µM	⇧p53 and ⇧p21/WAF1 ⇧Apoptosis via Fas/FasL apoptotic system Arrested at G1 phase (⇩Proliferation)	[90]
	A549	A549 IC_50_: 18.5 µM	⇧p21^CIP1/WAF^ via p53 independent pathway G2/M arrest(⇩Proliferation)	[91]
AML (acute myeloid leukemia)	HL-60	HL-60 ED_50_: 5.5 µg/mL (~21.46 µM) 5.00 µg/mL = 19.5 µM (72 h)	⇧DNA demethylation	[71]
	MV4-11 MOLM-13 OCI-LY10	MV4-11 IC_50_: 3.2 + 1.2 µM; MOLM-13 IC_50_: 4.9 + 2.1 µM OCI-LY10 IC_50_: 20.1 ± 6.7 µM (72 h)	⇧STAT5 ⇩FLT3/Erk1/2	[41]
	LCLs	Testing conc.: 0, 20, 40, 60, 80, 100, 120, 140 µM LCLs IC_50 estimated_: 40~65 µM (24 h) Applied 50 µM for studies.	⇧HMOX1, ⇧SLCO2B1, and⇧OKL38 ⇩CDK5R1 and CDC45L via p53 pathway	[84]
	HL-60	Testing conc.: 1~15 µg/mL (3.9 µM~58.54 µM) HL-60 IC_50 estimated_: ~40.42 µM (72 h)	⇧CD11b and ⇧CD14 expression (⇩Proliferation) ⇩iROS (⇧monocytic differentiation)	[85]
	RAW264.7	Testing conc.: 20 and 50 μM	⇩TRIF-dependent pathway ⇩NF-κB and ⇩IRF3	[92]
AML (acute myeloid leukemia)	RAW264.7	Testing conc.: 50 and 100 μM	⇧IRF3 ⇩TBK1 kinase activity ⇩IFNβ production	[93]
	HL-60	Testing conc.: 2.5~20 μg/mL (3.9 µM~78.05 µM) (Working conc.: 72 µM)	⇧CD11b and ⇧CD14 mRNA expression ⇧gp91phox and ⇧p47phox ⇧NADPH oxidase (⇩ROS) ⇩ROS (⇧HL-60 differentiation)	[94]
	HL-60	Testing conc.: 2.5~10 μg/mL (3.9 µM~39.0 µM)	⇧CD11b and ⇧CD14 (⇧Monocyte differentiation via Nrf2/ARE) ⇧Horseshoe-shaped nuclei ⇧Lipid peroxidation (MDA) level ⇩GSH/GSSG ratio (mRNA expression of ⇧CAT, ⇧NQO-1, ⇧Thioredoxin reductase and ⇧TRx)	[95]
	Jurkat J-Jhan J16 HUT78 Karpas 45	Jurkat IC_50_: 0.49 ± 0.12 nM (72 h) J-Jhan IC_50_: 1.55 ± 1.12 nM (72 h) J16 IC_50_: 5.25 ± 1.12 µM (72 h) HUT78 IC_50_: 11 ±13.5 µM (72 h) Karpas 45 IC_50_: 6.61 ± 1.07 µM (72 h)	ISL did not have a correlation with doxorubicin (DOX) and methotrexate (MTX) in genomic profiles. ISL is a valuable adjunct for cancer therapy, especially targeting on drug-resistant tumors.	[96]
	CCRF-CEM	CCRF-CEM IC_50_: 18.38 μM (24~72 h)	⇩Mitochondrial membrane potential disruption ⇧DNA damage G2/M arrest (⇩Proliferation) ⇩Cytochrome c	[97]
AML (acute myeloid leukemia)	Human monocyte model THP-1	N.A.	⇧DNCB-induced MAPK activation ⇧CD86 and ⇧CD54 ⇩DNCB-induced pro-inflammatory cytokines (⇩TNF-α, ⇩IL-6 and ⇩IL-4) ⇩p38-α and ⇩ERK activation	[98]
Melanoma	A375 A2058	Testing Conc: 0, 10, 20, 40, 80 µM A375 IC_50_: 21.63 µM (24 h) A2058 IC_50_: 20.75 µM (24 h)	⇧C-PARP, ⇧Bax, ⇧ cleaved-caspase-3(⇧Apoptosis) ⇩Proliferation ⇩Bcl-2	[99]
	B16F0	N.A.	⇧B16F0 differentiation	[100]
	A375	Testing Conc.: 0, 5, 10, 15 μg/mL (15 μg/mL = 58.53 µM) A375 IC_50 estimated_: ~48 µM	⇧Melanin content (⇧Melanogenesis) ⇧Tyrosinase (TYR) activity ⇧O_2_ consumption rate (OCR) G2/M cell cycle arrest ⇩mRNA level of GLUT1 and HK2 ⇩mTOR, ⇩*p*-mTOR, ⇩RICTOR, ⇩*p*-AKT, ⇩*p*-GSK3β	[101]
	A375	40 μg/mL: 69.86% 60 μg/mL: 92.22% A375 IC_50 estimated_: ~73 µM (24 h)	⇧Cleaved PARP and ⇧Cleaved caspase-3 ⇩Mitochondrial membrane potential ⇩mitoNEET	[102]
Melanoma	B16F0	Testing Conc.: 20, 40, 60 and 80 μg/mL B16F10 IC_50 estimated_: 35 μg/mL (~41.576 μM; 24 h) B16F10 IC_50 estimated_: 22 μg/mL (~86.77 μM; 48 h)	⇧ROS (⇧Apoptosis) Restart TCA cycle ⇩HIF-1α (Alleviating hypoxia) ⇩Lactate production ⇩Glucose uptake and glycolysis	[103]
	B16F10	Testing Conc.: 5, 10, 15, 20, and 25 μg/mL B16F10 IC_50 estimated_: ~19 μg/mL (~74.595 μM; 24 h) B16F10 IC_50 estimated_: ~10.5 μg/mL (~41.576 μM; 48 h)	⇧TYR Activity ⇧Melanin Biosynthesis ⇧ROS ⇩Colony formation ⇩Cell proliferation	[104]
	ARH-77 U266 MPC-11 SP2/0 CZ-1 RPMI8226	ARH-77 IC_50_: ~13.54 µM MPC-11 IC_50_: ~4.45 µM SP2/0 IC_50_: ~22.91 µM CZ-1 IC_50_: ~13.93 µM U266 IC_50_: ~8.62 µM RPMI8226 IC_50_: ~9.09 µM IC_50_ of ISL was < 4 μg/mL (48 h)	⇧Cleavage caspase-3 ⇩IL-6 ⇩p-ERK and ⇩*p*-STAT3 ⇩Bcl-2, ⇩Bcl-XL and ⇩pro-caspase-3	[105]
	SK-MEL-2 HaCaT	Testing Conc.: 0, 1, 4, and 8 µM SK-MEL-2 cells and HaCaT cells (48 h) treated less than 8 µM showed no cytotoxic effects	⇧p-p38 ⇩Tyrosinase (⇩Tyrosine kinase) ⇩ TRP-1, ⇩DCT, ⇩Rab27a and ⇩Cdc42 ⇩ ERK pathway (⇩Degradation of MITF)	[106]
Melanoma	B16 mouse melanoma 4A5 cells	Testing 150 and 200 µM (18 and 24 h)	⇧Apoptosis (p53 independent pathway) ⇧Bax ⇩Cell proliferation ⇩Glucose transmembrane transport	[107]
HCC/Hepato-ma	Hep3B	Hep3B IC_50_: 42.84 + 2.01 μM 50 μM applied (48 h)	⇧P21, ⇧P27 G1/S cell cycle arrest (⇩Proliferation) ⇩Cyclin D1 ⇩PI3K/AKT pathway ⇧E-cadherin, ⇩Vimentin and ⇩N-cadherin (⇩Migration and ⇩metastasis)	[108]
	HepG2 Hep3B	Testing conc.: 20, 40, 60, 80, and 100 μM (18 h) HepG2 IC_50_: 27.71 μM Hep3B IC_50_: 35.28 μM	⇧ MAPK/STAT3/NF-κB (⇧Apoptosis) ⇧ ROS accumulation ⇧Phosphorylated c-Jun N-terminal kinase (JNK), ⇧P21, ⇧p38 kinase G2/M arrest (⇩Proliferation) ⇩p-ERK, ⇩p-STAT3, and ⇩NF-κB (p65) ⇩Cyclin B1, ⇩CDK1/2, and ⇩p27	[109]
	HepG2	Testing conc.: 1, 5, 10, 20 μg HepG2 IC_50 estimated_: ~88.46 μM (24 h) HepG2 IC_50 estimated_: ~31.07 μM (48 h)	⇧p53, ⇧p21/WAF1, ⇧ Fas/APO-1 receptor, Fas ligand, ⇧Bax and ⇧NOXA (⇧Chemopreventive effect) G2/M-phase arrest	[110]
	HepG2	HepG2 IC_50_: 10.51 μg/mL (~39 μM; 48 h)	⇧IkB ⇩NF-κB, Bcl-X_L_, c-IAP1/2	[111]
HCC/Hepato-ma	SNU475	SNU475 IC_50_: 0.243 + 0.21 mM	⇩ DNA cleavage reaction (Stabilized DNA) ⇩TOP I activity(ISL-TOP I interaction: 0.18 + 0.12 mM)	[50]
	Hepa 1c1c7	Hepa 1c1c7 IC_50_: 36.3 μM	ISL is a chemopreventive reagent	[112]
	Hep3B	Hep3B IC_50_: 50.8 μM	⇩CK2 activity (CK2 IC_50_: 17.3 uM)	[45]
	SK-Hep-1	SK-Hep-1 IC_50:_ 19.08 μM	⇩ Proliferation	[113]
	PC-3 22RV1	Testing conc: 0, 1, 10, 25, 50, and 100 μM) PC-3 IC_50_: 19.6 μM (48 h) 22RV1 IC_50_: 36.6 μM (48 h)	⇧Apoptosis G2/M cell cycle arrest ⇩Cyclin B1, ⇩CDK1 (p-Thr14, p-Tyr15, and p-Thr161)	[114]
Prostate cancer	C4-2 LNCaP IEC-6	10~100 μM (24 h) C4-2 IC_50_: 87.0 μM	⇧AMPK and ⇧pERK (⇩Proliferation) ⇧*p*-p38 ⇩Psi(m) (⇧Apoptosis)	[59]
	DU145	Applied conc.: 5~20 μM	⇧*p*-CDC2 (Tyr15) and ⇧Cyclin B1 ⇧G1 phase ⇧p27^KIP1^ G2/M cell cycle arrest ⇩CDC25C	[115]
	DU145	Applied conc.: 0~20 μM	⇩JNK/AP-1 signaling ⇩VEGF, ⇩integrin-α2, ⇩ICAM and ⇩VCAM ⇩Invasion and ⇩metastasis via ⇩µPA, ⇩MPP-9 and ⇩AP-1	[116]
	DU145	Applied conc.: 0~20 μM	⇩PI3K/AKT and ErbB3 pathway (⇩Proliferation) ⇩HRG-β-induced ErbB3 signaling (⇩ErbB3)	[117]
Prostate cancer	MAT-LyLu DU145	Applied conc.: 0~20 μM MAT-LyLuIC_50 estimated_: ~13.74/5.67/5.01 µM DU145 IC_50 estimated_: ~56.87/31.49/17.60 µM (24 h/48 h/72 h)	⇧ Fas ligand (FasL), ⇧Fas, ⇧Cleaved caspase-8 and ⇧tBid (⇧Apoptosis) lic>249) ⇧Cytochrome c and Smac/Diablo	[118]
	DU145 LNCaP	Testing conc.: 0, 5, 10, 15, and 20 μM DU145 IC_50 estimated_: ~10.561 µM (48 h) LNCaP IC_50 estimated_: ~10.775 µM (48 h)	⇧GADD153 mRNA S and G2/M arrest	[119]
Cervical cancer	Ca Ski SiHa HeLa C-33A	Testing conc: 10, 20, 40, and 80 µM Ca Ski IC_50 estimated_: 39.09 μM (72 h) SiHa IC_50 estimated_: 53.76 μM (72 h) HeLa IC_50 estimated_: 58.10 μM (72 h) C-33A IC_50 estimated_: 32.83 μM (72 h)	⇧p53, ⇧p21, ⇧Bax ⇧Cleavage of caspase-9, ⇧caspase-3, ⇧PARP and ⇧caspase -8 ⇩Bcl-2	[120]
	HeLa	Testing conc: 2, 5, 10, 30, 40, and 60 μg/mL HeLa IC_50 estimated_: ~21.24 μM (24 h)	⇧ROS ⇧*p*-eIF2α, ⇧GRP78 level (⇧ER stress) ⇧Caspase-12 G2/M cell cycle arrest (⇩Proliferation) ⇩Bcl-2	[121]
	HeLa	HeLa IC_50_: 9.8 μM (48 h)	⇧p53 ⇧*p*-Chk2, ⇧*p*-cdc25C, and ⇧*p*-cdc2 G2/M cell cycle arrest ⇩*p*-p53 (Serine15) ⇩Bcl-2, Bcl-XL ⇩Cyclin B, ⇩cyclin A, ⇩cdc2, and ⇩cdc25C	[122]
Gastric cancer	MKN28	MKN28 IC_50_: ~20.84 µM (48 h)	⇧Beclin 1 ⇩p62 (⇧Autophagy) ⇩*p*-AKT and ⇩*p*-TOR (⇧Apoptosis)	[123]
	MKN-45	5 µM applied	⇩H2R and ⇩c-Fos/c-Jun	[46,124]
	MGC-803	0.11 g/L applied (24 h)	Calcium- and delta psi(m)-dependent (⇧Apoptosis)	[125]
	SGC-7901 BGC-823	BGc-823 IC_50_: 23.18 µM (48 h) SGC-7901 IC_50_: 12.91 µM (48 h)	⇧G2/M cell cycle arrest (⇩Proliferation) ⇧Cleaved-PARP, ⇧Bcl-2 and ⇧Bax (⇧Apoptosis) ⇧LC3B II and⇧ Beclin 1(⇧Autophagy) ⇩PI3K/AKT/mTOR	[32]
Uterine leiomyoma	Leiomyma Myomentrium	Testing conc: 0, 10, 20, 50 µM Leiomyma IC_50 estimated_ = ~39.33 µM Myomentrium IC_50 estimated_ = ~698.8 µM (48 h)	⇧FAS ligand expression(⇧Apoptosis) ⇧p21^Cip1/ Waf^ (⇧Apoptosis via p53-dependent) ⇧Caspase-3 activation subG1 and G2/M arrest (⇩Proliferation) ⇩Bcl-2, ⇩cdk 2/4, and ⇩E2F	[126]
Osteosarcoma	U2OS	Testing conc: 5, 10, and 20 µM 20 μM applied	⇧Bax and ⇧caspase-3 (⇧Apoptosis) ⇧p53, ⇧p21 and ⇧p27 ⇩Bcl2,⇩PI3K/AKT/mTOR pathway ⇩p70, ⇩Cyclin D1, ⇩Bcl‑2, ⇩MMP-2/⇩MMP-9	[127,128]
	Saos‑2 MC3T3-E1	Saos‑2 IC_50 estimated_ = ~24.23 μM 30 μM applied		
Glioma	SK-N-BE(2) IMR-32	Effective conc. > 5 µM	⇧ROS (⇧Necrosis)	[129]
	U87	U87 IC_50_: 6.3 µM	⇧Caspase-3 ⇩TOP I	[130]
	PC12	PC12 IC_50_: 17.8 ± 1.8 μM	⇧Caspase-9, ⇧caspase-3, ⇧ caspase-7, ⇧Bax, ⇧Bim, and ⇧cytochrome c (⇧Apoptosis) ⇧Beclin-1 and ⇧LC3 (⇧Autophagy) ⇩Bcl-2 and ⇩Bcl-x	[131]
Bladder cancer	T24	Effective conc.: 30 and 70 µg/mL (24 h)	⇧Bax, ⇧Bim, ⇧Apaf-1, ⇧Caspase-9, ⇧Caspase-3, and ⇧CDK2 activity ⇩ΔΨm and ⇩Bcl-2	[132]
Oral squamous cell carcinomas (OSCC)	SG SAS-CSCs OECM-1	SG cells IC_50_: 386.3 ± 29.7 μM SAS-CSCs IC_50_: 144.9 ± 25.7 μM OECM-1-CSCs IC_50_: 104.5 ± 26.2 μM	⇩GRP78 ⇩CSCs properties ⇩ABCG2 expression	[60]

Note: The ‘’IC_50_ estimated’’ indicated Data extracted from published figures using Web Plot Digitizer (https://automeris.io/WebPlotDigitizer), then analyzed IC_50_ by “Quest Graph™ IC_50_ Calculator.” AAT Bioquest Inc, 27 October 2020, https://www.aatbio.com/tools/ic50-calculator [133].

**Table 4:** In vivo model demonstrated the ISL induced different pathway in various cancers.

Cancer	Tumor Model	Dose	Applied	Duration	Effect of ISL In Vivo	Ref
Breast cancer	MDA-MB-231 bearing female nude mice	20 mg/kg/day 50 mg/kg/day	IP	25 days	ISL inhibit angiogenesis ISL inhibit breast cancer growth Little influence on normal tissue	[57]
	MDA-MB-231 bearing Balb/c nu/nu mice	10 mg/kg/day 20 mg/kg/day 5 times/week	Oral	38 days	Anti-metastatic activities	[64]
	6-week-old female, MDA-MB-231 bearing BALB/c nude mice	50 mg/kg/day 100 mg/kg/day 3 times/week	IP	5 weeks	Cancer growth inhibition and through downregulating AA metabolic network and the deactivation of PI3K/AKT in human breast cancer	[66]
	MMTV-PyMT transgenic mice	50 mg/kg/day	Oral	7 weeks (4th~11th week)	Suppress cancer growth and inhibit the metastasis via regulating miR-374a/PTEN/AKT axis Little influence on normal tissue	[67]
	MMTV-PyMT mice	50 mg/kg/day	Oral	8~12 weeks (4th~12nd or 15th week)	ISL treatment significantly limited tumor foci growth and dispersion by promoting the demethylation of WIF1 promoter	[51]
	4-week-old female NOD/SCID mice bearing MDA-MB-231	50 mg/kg/day	Oral	4 weeks	Chemosensitize breast CSCs via inhibiting the GRP78/β-catenin/ABCG2 pathway	[40]
	4T1-bearing nude-mouse model	25 mg/kg/2 days	IP	20 days	iRGD modified lipid–polymer hybrid NPs improve the efficacy of ISL in anti-breast cancer	[22]
	5-week-old female nude-Foxn1^nu^ mice bearing MDA-MB-231	2.5–5 mg/mL 0.25 mL/day	Oral gavage	14~25 days	Inhibit triple-negative breast cancer cell (MDA-MB-231) growth through autophagy-mediated apoptosis	[69]
	MCF-7aro xenograft model	50~150 ppm or 0.15~0.5% in diet	Oral in diet	13th~77th days	ISL acts as a chemoprotective agent to inhibit the enzyme and transcriptional activity of CYP19	[70]
Colon	7–8 week-old male BALB/c nude mice bear HCT116 tumor	2.5 mg/kg/2days 5 mg/kg/2days	Peritumoral injection	14 days	Mediate apoptotic through p62/SQSTM1 upregulation in CRC cancer	[73]
	6-week-old male ddY AOM induced mice	10 ppm 100 ppm 250 ppm	In drinking water	16~24 weeks	Against colon cancer	[134]
	BALB/c male mice bearing CT26	1 mg/kg/day 5 mg/kg/day	PO IP	15 days	Inhibited the growth of tumors ISL alleviates cisplatin-induced nephrotoxicity and hepatotoxicity Improved the side effects of cisplatin therapy	[75]
	Male AOM-treated F344 rats	100 ppm mixed in MF basal diet	Oral	4 weeks	Inhibited the induction of preneoplastic aberrant crypt foci (ACF) ISL is a promising chemopreventive agent against colon carcinogenesis	[76]
	6-week-old DSS-induced colitis mice	30 mg/kg	Oral	10 days	Inhibited MAPK pathway and suppressed the phosphorylation of ERK1/2 and p38, and the activation of NK-κB in colon tissue	[135]
	NOD-SCID old female mice	25 mg/kg, 50 mg/kg 100 mg/kg	Oral	18~30 days observed~80 days	Anti-AML via ISL direct interact with FLT3 kinase (IC_50_ value of 115.1 ± 4.2 nM)	[41]
AML	7~8-week-old female NOD-SCID mice bearing MV4-11 cells (AML xenograft model)	25 mg/kg/day 50 mg/kg/day 100 mg/kg/day	Oral	30 days	ISL significantly inhibited the MV4-11 flank tumor growth and prolonged survival in the bone marrow transplant model via decreasing the expression of Ki67 and inducing apoptosis	[41]
Immuno- response	6–8-week-old male and female BALB/c mice (AD-like lesion model)	1% ISL daily	Oral	6th~18th (12 days)	ISL significantly suppressed the DNCB-induced IgE and Th2 cytokines up-regulation	[98]
	DTH animal model with IKKβC46A transgenic (IKKβC46A in C57BL/6 mice)	0.75 mg/ear		24, 48, 72 h	ISL inhibited T cell activation in vivo via directly binding to IKKβ Cys46	[43]
Lung	Carrageenan-induced pleurisy mice model	30 mg/kg	IP	Twice a day (12 h)	Activation of Nrf2 pathway thus decreasing oxidative stress Inhibition of the NF-κB, MAPK and NLRP3 pathways (with high level of iNOS and COX-2) causes anti-inflammatory activities	[136]
	CS-induced COPD mice	10 mg/kg 20 mg/kg 30 mg/kg	Oral	Twice a day for 4 weeks	ISL inhibit inflammatory and oxidative stress via the regulation of the Nrf2 and NF-κB signaling pathways	[137]
	LPS-induced acute lung injury (ALI) in male BALB/c mice	5 mg/kg 10 mg/kg 20 mg/kg	Intracheal instillation	Twice a day (12 h)	ISL inhibited the inflammatory of LPS-induced lung injury by activating PPAR-γ and inhibiting NF-κB activation	[138]
	Pulmonary metastasis model: BALB/c mouse bearing Renca cells	0.1, 0.5, 2 and 10 mg/day	IP	10 days	ISL prevented severe leukocytopenia caused by administration of 5-FU	[139]
Lung	LPS-Induced ALI mouse model in C57BL/6 mice	30 mg/kg	IP	A single dose	ISL treatment significantly alleviated lung injury in LPS-induced ALI mice via activating AMPK/Nrf2/ARE signaling and inhibited LPS-induced NLRP3 and NF-κB pathway	[86]
	6~8-week-old C57BL/6 mice (Influenza virus infected model)	10 mg/kg	IP	18 days	ISL is a dual PPARγ and Nrf2 agonist with antiviral and anti-inflammatory properties that protect against influenza virus infection	[87]
	6-week-old Athymic nude mice bearing NCI-H1975 cells	1 mg/kg 5 mg/kg	IP	Three times per week, 12 days	ISL suppresses NSCLC cell growth by directly targeting wild type or mutant EGFR. Anticancer effects of ISL in NSCLC cells modulated the EGFR signaling through downstream AKT and ERK1/2	[42]
	Induce tracheal relaxation model in male Hartley guinea-pigs	5 mg/kg 10 mg/kg 20 mg/kg	IG intraduodenal	A single dose	ISL activated the cGMP/PKG signaling cascade through PKG-dependent mechanism and thus to tracheal relaxation	[89]
Melanoma	8-week-old immunocompromised mice bearing A2058	20 mg/kg	IP every other days	42 days	ISL may inhibit the proliferation of melanoma cells by suppressing miR-301b and inducing its target LRIG1	[99]
	6~8-week-old male C57BL/6 mice bearing B16F0 melanoma cells	15 μg/mL	Oral	48 h	ISL-induced differentiation of B16F0 cells accompanied increased ROS formation	[104]
	4~5-week-old female SCID mice bearing U266 and male BABL/c bearing MPC-11 tumor	100 μg/kg/day 200 μg/kg/day	IP	15~20 days	ISl mediated IL-6 signaling	[105]
HCC	4~5w-week-old female BALB/c- mice bearing Hep3B cells	50 mg/kg/day	IP	3 weeks	ISL can prevent HCC tumorigenesis and metastasis through suppressing cyclin D1 and PI3K/AKT pathway	[108]
	4-week-old male athymic BALB/c nude mice bearing HepG2	10 mg/kg	IP	A single dose	The effects of ISL on radiosensitization via Nrf2⇩-Keap1⇩ pathway	[140]
Prostate cancer	6-week-old male BALB/c nude mice bearing PC-3	25 mg/kg/day 50 mg/kg/day	IP	~28 days	IISL modulates cyclin B1–CDK1 for G2/M arrest and apoptosis	[114]
Ovary cancer	6-week-old female athymic nude mice were intraperitoneally injected SKOV3 cells	12.5 mg/kg 25 mg/kg	IP every other days	3 weeks	ISL at a noncytotoxic concentration was able to antagonize EMT ISL blocks ovarian cancer EMT by interfering with the TGF-pathway	[79]
Gastric cancer	Xenograft NOD/SCID mice bearing EBV(+) or EBV(−) human gastric carcinoma (SNU719 or MKN74)	30 mg/kg/day	Oral	2 weeks	ISL have anti-tumor effects through up-regulating the expressions of p53, Bax, and Puma and the cleaved forms of Caspase-3 and -9 and Parp protein	[124]
Osteosarco-ma	5-week-old female NOD-SCID mice bearing Saos-2	50 mg/kg/day	Oral gavage	56 days	ISL inhibit cell proliferation and induce the cell apoptosis via deactivating the PI3K/AKT signaling pathway	[128]
Oral cancer	5–6 week-old nude mice (BALB/c nu/nu mice) bearing OSCC-CSCs	5 mg/kg/day	Oral gavage	20 days	ISL-mediated reduction of GRP78 in OSCC-CSCs played a critical role	[131]

**Table 5:** ISL combined with other cancer treatment.

Type of Cancer	IC_50_	Combination Treatment	Combination Effect	Ref
	In Vitro/In Vivo			
Breast cancer	In vitro: MCF-7, MDA-MB-231 In vivo: NOD/SCID mice bearing MDA-MB-231 or MCF-7/ADR	ISL + 5-FU ISL + epirubicin ISL + taxol	ISL possess chemosensitizing effects via activation of autophagy ISL limited the self-renewal and differentiation abilities of breast CSCs via GRP78/β-catenin/ABCG2 signaling	[40,56]
Colon cancer	In vitro: HT29	ISL + TRAIL	ISL up-regulates a TRAIL receptor DR5 protein overcomes TRAIL resistance in colon cancer	[72]
	In vitro: HTC116	ISL + 5-FU	ISL-induced p62/SQSTM1 expression mediated apoptosis by reducing caspase-8 activation	[73]
	In vivo: CT26 murine colon cancer cells	ISL + cisplatin	ISL reduced tumor sizes without any detectable nephrotoxicity or hepatoxicity. ISL suppressed cisplatin-induced kidney and liver damage led to a syngeneic effect for anti-cancer	[75]
	In vitro: CEM/ADR 5000 cells and Caco-2 cells	ISL + doxorubicin ISL + doxorubicin+ saponin digitonin	In combined therapy, ISL was identified as potential multidrug resistance (MDR) modulator which serves as a chemo-adjuvant therapy	[11]
Melanoma	In vivo: MM xenograft models	ISL + *adriamycin*	ISL could inhibit the growth of MM via blocking IL-6 ISL synergistically enhanced the anti-myeloma activity of adriamycin	[105]
Liver cancer	In vitro: HepG2 In vivo: BALB/c bearing HepG2	ISL + Radiochemotherapy	ISL induced oxidative stress (ROS) by disturbing the redox status and ultimately enhancing the radiosensitivity ISL on radiosensitization via Nrf2-Keap1 pathway	[111,143]
				[57,140]
Cervical cancer	In vitro: HeLa cell	ISL + ROS scavengers	ISL induced apoptosis by increasing intracellular ROS levels	[144]
	In vivo: KM mice bearing U14	ISL + cyclophosphamide	ISL enhanced antitumor activity of CP in vivo and decreased the micronucleus formation DNA strand breaks	[145]
Gastric cancer	In vitro: MKN45	ISL + 5-FU	ISL downregulated GRP78 and CSCs- marker, ABCG2, LGR5, CD24 and CD44 to enhance chemosensitivity with combination of 5-FU	[146]
Leukemia	In vitro: T-ALL cells	ISL + DOX ISL + MTX	ISL may be a valuable adjunct for cancer therapy to treat otherwise drug-resistant tumors	[96]
Lung cancer	Pulmonary metastasis model: BALB/c mouse bearing Renca cells	ISL + 5-FU	ISL suppressed tumor proliferation, potentiated nitric oxide production by lipopolysaccharide-stimulated macrophages, and facilitated cytotoxicity of splenic lymphocytes in vitro	[139]
Asthma	In vitro: D10 cells In vivo: OVA sensitization/ 7, 4′-DHF challenge	ISL + ASHMI™	ISL increased IFN-γ expression involving anti-inflammatory effect ISL reduced eosinophilic pulmonary inflammation via suppressed Th2 cytokines, IL-4 and IgE production	[147]
Oral cancer	Oral squamous cell carcinomas In vivo: nude mice bearing OSCC	ISL + cisplatin	ISL mediated GRP78 regulation serves as chemotherapy adjuvant	[60]
Bladder cancer	T24	ISL + cisplatin	ISL treatment with cisplatin increases cell death in bladder cancer cells	[148]
Uterine sarcoma	MES-SA/Dx5, MES-SA/Dx5-R	ISL + doxorubicin	ISL enhanced chemosensitivity via inducing apoptosis and autophagy ISL inhibits mTOR pathway	[142]
Kidney cancer	LLC-PK1	ISL + cisplatin	ISL pretreatment induces ER stress and produces hormesis to protect against CP-induced nephrotoxicity	[149]
Neuroblastoma	In vitro: MYC-amplified NB cells SK-N-BE(2) and IMR-32	ISL + cisplatin	Treated ISL with cisplatin resulted in loss of cell viability greatly, acting as a potential adjunct therapy	[129]

## Data Availability

All the data presented in this study are included in this article.

## References

[1] Bray F., Ferlay J., Soerjomataram I., Siegel R.L., Torre L.A., Jemal A. (2018). Global cancer statistics 2018: GLOBOCAN estimates of incidence and mortality worldwide for 36 cancers in 185 countries. Cancer J. Clin..

[2] Panda A.K., Chakraborty D., Sarkar I., Khan T., Sa G. (2017). New insights into therapeutic activity and anticancer properties of curcumin. J. Exp. Pharmacol..

[3] Walker B.R., Edwards C.R. (1994). Licorice-induced hypertension and syndromes of apparent mineralocorticoid excess. Endocrinol. Metab. Clin. N. Am..

[4] Lee S.K., Park K.-K., Park J.H.Y., Lim S.S., Chung W.-Y. (2013). The Inhibitory Effect of Roasted Licorice Extract on Human Metastatic Breast Cancer Cell-Induced Bone Destruction. Phytother. Res..

[5] Guo J., Liu D., Nikolic D., Zhu D., Pezzuto J.M., Van Breemen R.B. (2007). In Vitro Metabolism of Isoliquiritigenin by Human Liver Microsomes. Drug Metab. Dispos..

[6] Cuendet M., Guo J., Luo Y., Chen S.-N., Oteham C.P., Moon R.C., Van Breemen R.B., Marler L.E., Pezzuto J.M. (2010). Cancer Chemopreventive Activity and Metabolism of Isoliquiritigenin, a Compound Found in Licorice. Cancer Prev. Res..

[7] Guo J., Liu A., Cao H., Luo Y., Pezzuto J.M., Van Breemen R.B. (2008). Biotransformation of the Chemopreventive Agent 2′,4′,4-Trihydroxychalcone (Isoliquiritigenin) by UDP-Glucuronosyltransferases. Drug Metab. Dispos..

[8] Yang E.-J., Kim M., Woo J.E., Lee T., Jung J.-W., Song K.-S. (2016). The comparison of neuroprotective effects of isoliquiritigenin and its Phase I metabolites against glutamate-induced HT22 cell death. Bioorg. Med. Chem. Lett..

[9] Chen C., Shenoy A.K., Padia R., Fang D.-D., Jing Q., Yang P., Shi-Bing S., Huang S. (2018). Suppression of lung cancer progression by isoliquiritigenin through its metabolite 2, 4, 2’, 4’-Tetrahydroxychalcone. J. Exp. Clin. Cancer Res..

[10] Lee Y.K., Chin Y.-W., Bae J.-K., Seo J.S., Choi Y.H. (2013). Pharmacokinetics of Isoliquiritigenin and Its Metabolites in Rats: Low Bioavailability Is Primarily Due to the Hepatic and Intestinal Metabolism. Planta Med..

[11] Zhou J.-X., Wink M. (2018). Reversal of Multidrug Resistance in Human Colon Cancer and Human Leukemia Cells by Three Plant Extracts and Their Major Secondary Metabolites. Medicines.

[12] Yushan R., Ying Y., Yujun T., Jingchun Y., Dongguang L., Lihong P., Pingping W., Lili Z., Fanhui Z., Zhong L. (2018). Isoliquiritigenin inhibits mouse S180 tumors with a new mechanism that regulates autophagy by GSK-3beta/TNF-alpha pathway. Eur. J. Pharmacol..

[13] Han B., Chen W., Zheng Q., Wang X., Yan H., Li L., Aisa H. (2011). Determination of Isoliquiritigenin and Its Distribution in Mice by Synchronous Fluorescence Spectrometry. Anal. Sci..

[14] Qiao H., Zhang X., Wang T., Liang L., Chang W., Xia H. (2014). Pharmacokinetics, biodistribution and bioavailability of isoliquiritigenin after intravenous and oral administration. Pharm. Biol..

[15] Li H., Ye M., Zhang Y., Huang M., Xu W., Chu K., Chen L., Que J. (2012). Blood-brain barrier permeability of Gualou Guizhi granules and neuroprotective effects in ischemia/reperfusion injury. Mol. Med. Rep..

[16] Ma X., Fang F., Song M., Ma S. (2015). The effect of isoliquiritigenin on learning and memory impairments induced by high-fat diet via inhibiting TNF-α/JNK/IRS signaling. Biochem. Biophys. Res. Commun..

[17] Zhang K., Wang Q., Yang Q., Wei Q., Man N., Adu-Frimpong M., Toreniyazov E., Ji H., Yu J., Xu X. (2019). Enhancement of Oral Bioavailability and Anti-hyperuricemic Activity of Isoliquiritigenin via Self-Microemulsifying Drug Delivery System. AAPS PharmSciTech.

[18] Liu J., Wang Q., Adu-Frimpong M., Wei Q., Xie Y., Zhang K., Wei C., Weng W., Ji H., Toreniyazov E. (2019). Preparation, in vitro and in vivo evaluation of isoliquiritigenin-loaded TPGS modified proliposomes. Int. J. Pharm..

[19] Zhang X., Zhang T., Shi Y., Ni J. (2014). Enhancement of gastrointestinal absorption of isoliquiritigenin by nanostructured lipid carrier. Adv. Powder Technol..

[20] Qiao F., Zhao Y., Mai Y., Guo J., Dong L., Zhang W., Yang J. (2020). Isoliquiritigenin Nanosuspension Enhances Cytostatic Effects in A549 Lung Cancer Cells. Planta Med..

[21] Sun X., Zhang J., Wang Z., Liu B., Zhu S., Zhu L., Peng B. (2019). Licorice isoliquiritigenin-encapsulated mesoporous silica nanoparticles for osteoclast inhibition and bone loss prevention. Theranostics.

[22] Gao F., Zhang J., Fu C., Xie X., Peng F., You J., Tang H., Wang Z., Li P., Chen J. (2017). iRGD-modified lipid-polymer hybrid nanoparticles loaded with isoliquiritigenin to enhance anti-breast cancer effect and tumor-targeting ability. Int. J. Nanomed..

[23] Zhang X., Qiao H., Chen Y., Li L., Xia H., Shi Y. (2016). Preparation, properties and preclinical pharmacokinetics of low molecular weight heparin-modified isoliquiritigenin-loaded solid lipid nanoparticle. Iran. J. Pharm. Res..

[24] Zhang X.-Y., Qiao H., Ni J.M., Shi Y.B., Qiang Y. (2013). Preparation of isoliquiritigenin-loaded nanostructured lipid carrier and the in vivo evaluation in tumor-bearing mice. Eur. J. Pharm. Sci..

[25] Noh G.Y., Suh J.Y., Park S.N. (2017). Ceramide-based nanostructured lipid carriers for transdermal delivery of isoliquiritigenin: Development, physicochemical characterization, and in vitro skin permeation studies. Korean J. Chem. Eng..

[26] Xie Y.-J., Wang Q., Adu-Frimpong M., Liu J., Zhang K.-Y., Xu X., Yu J.-N. (2019). Preparation and evaluation of isoliquiritigenin-loaded F127/P123 polymeric micelles. Drug Dev. Ind. Pharm..

[27] Wang G., Yu Y., Wang Y.-Z., Yin P.-H., Xu K., Zhang H. (2020). The effects and mechanisms of isoliquiritigenin loaded nanoliposomes regulated AMPK/mTOR mediated glycolysis in colorectal cancer. Artif. Cells Nanomed. Biotechnol..

[28] Kong B.J., Kim A., Park S.N. (2016). Properties and in vitro drug release of hyaluronic acid-hydroxyethyl cellulose hydrogels for transdermal delivery of isoliquiritigenin. Carbohydr. Polym..

[29] Cao M., Zhan M., Wang Z., Wang Z., Li X.-M., Miao M. (2020). Development of an Orally Bioavailable Isoliquiritigenin Self-Nanoemulsifying Drug Delivery System to Effectively Treat Ovalbumin-Induced Asthma. Int. J. Nanomed..

[30] Jing Z., Ji-Liang W., Lin Z., Chun Z. (2004). Preparation of isoliquiritigenin liposome and its inhibitive effects on proliferation of human cervical cancer cells in vitro. Chin. J. Clin. Pharmacol. Ther..

[31] Peng F., Meng C.-W., Zhou Q.-M., Chen J.-P., Xiong L. (2015). Cytotoxic Evaluation against Breast Cancer Cells of Isoliquiritigenin Analogues from Spatholobus suberectus and Their Synthetic Derivatives. J. Nat. Prod..

[32] Huang F., Wang J., Xu Y., Zhang Y., Xu N., Yin L. (2020). Discovery of novel isoliquiritigenin analogue ISL-17 as a potential anti-gastric cancer agent. Biosci. Rep..

[33] Peng F., Xiong L., Xie X., Tang H., Huang R., Peng C. (2020). Isoliquiritigenin Derivative Regulates miR-374a/BAX Axis to Suppress Triple-Negative Breast Cancer Tumorigenesis and Development. Front. Pharmacol..

[34] Jeong S., Lee S., Kim K., Lee Y., Lee J., Oh S., Choi J.W., Kim S.W., Hwang K.C., Lim S. (2020). Isoliquiritigenin Derivatives inhibit RANKL-induced osteoclastogenesis by regulating p38 and NF-κB activation in RAW 264.7 cells. Molecules.

[35] Gaur R., Yadav K.S., Verma R.K., Yadav N.P., Bhakuni R.S. (2014). In vivo anti-diabetic activity of derivatives of isoliquiritigenin and liquiritigenin. Phytomedicine.

[36] Selvaraj B., Kim D.W., Huh G., Lee H., Kang K., Lee J.W. (2020). Synthesis and biological evaluation of isoliquiritigenin derivatives as a neuroprotective agent against glutamate mediated neurotoxicity in HT22 cells. Bioorg. Med. Chem. Lett..

[37] Reddy M.R., Aidhen I.S., Reddy U.A., Reddy G.B., Ingle K., Mukhopadhyay S., Ingle K. (2019). Synthesis of 4-C -β-D-Glucosylated Isoliquiritigenin and Analogues for Aldose Reductase Inhibition Studies. Eur. J. Org. Chem..

[38] Gay N.H., Suwanjang W., Ruankham W., Songtawee N., Wongchitrat P., Prachayasittikul V., Prachayasittikul S., Phopin K. (2020). Butein, isoliquiritigenin, and scopoletin attenuate neurodegeneration via antioxidant enzymes and SIRT1/ADAM10 signaling pathway. RSC Adv..

[39] Wang Z., Wang N., Han S., Wang D., Mo S., Yu L., Huang H., Tsui K., Shen J., Chen J. (2013). Dietary Compound Isoliquiritigenin Inhibits Breast Cancer Neoangiogenesis via VEGF/VEGFR-2 Signaling Pathway. PLoS ONE.

[40] Wang N., Wang Z., Peng C., You J., Shen J., Han S., Chen J. (2014). Dietary compound isoliquiritigenin targets GRP78 to chemosensitize breast cancer stem cells via beta-catenin/ABCG2 signaling. Carcinogenesis.

[41] Cao Z.-X., Wen Y., He J.-L., Huang S.-Z., Gao F., Guo C.-J., Liu Q.-Q., Zheng S.-W., Gong D.-Y., Li Y.-Z. (2019). Isoliquiritigenin, an Orally Available Natural FLT3 Inhibitor from Licorice, Exhibits Selective Anti-Acute Myeloid Leukemia Efficacy In Vitro and In Vivo. Mol. Pharmacol..

[42] Jung S.K., Lee M.-H., Lim D.Y., Kim J.E., Singh P., Lee S.-Y., Jeong C.-H., Lim T.-G., Chen H., Chi Y.-I. (2014). Isoliquiritigenin Induces Apoptosis and Inhibits Xenograft Tumor Growth of Human Lung Cancer Cells by Targeting Both Wild Type and L858R/T790M Mutant EGFR. J. Biol. Chem..

[43] Yan F., Yang F., Wang R., Yao X.J., Bai L., Zeng X., Huang J., Wong V.K., Lam C.W., Zhou H. (2017). Isoliquiritigenin suppresses human T Lymphocyte activation via covalently binding cysteine 46 of IkappaB kinase. Oncotarget.

[44] Park S.J., Youn H.S. (2010). Isoliquiritigenin suppresses the Toll-interleukin-1 receptor domain-containing adapter inducing interferon-beta (TRIF)-dependent signaling pathway of Toll-like receptors by targeting TBK1. J. Agric. Food Chem..

[45] Qi X., Zhang N., Zhao L., Hu L., Cortopassi W.A., Jacobson M.P., Li X., Zhong R. (2019). Structure-based identification of novel CK2 inhibitors with a linear 2-propenone scaffold as anti-cancer agents. Biochem. Biophys. Res. Commun..

[46] Kim D.-C., Choi S.-Y., Kim S.-H., Yun B.-S., Yoo I.-D., Reddy N.R.P., Yoon H.S., Kim K.-T. (2006). Isoliquiritigenin Selectively Inhibits H2 Histamine Receptor Signaling. Mol. Pharmacol..

[47] Wang C., Chen L., Cai Z.C., Chen C., Liu Z., Liu X., Zou L., Chen J., Tan M., Wei L. (2020). Comparative Proteomic Analysis Reveals the Molecular Mechanisms Underlying the Accumulation Difference of Bioactive Constituents in Glycyrrhiza uralensis Fisch under Salt Stress. J. Agric. Food Chem..

[48] Khan S.I., Zhao J., Ibrahim M., Walker L.A., DasMahapatra A.K. (2011). Potential utility of natural products as regulators of breast cancer-associated aromatase promoters. Reprod. Biol. Endocrinol..

[49] Shah U., Patel S., Patel M., Gandhi K., Patel A. (2020). Identification of chalcone derivatives as putative non-steroidal aromatase inhibitors potentially useful against breast cancer by molecular docking and ADME prediction. Indian J. Chem. Sect. B.

[50] Li Z.-X., Li J., Li Y., You K., Xu H., Wang J. (2015). Novel insights into the apoptosis mechanism of DNA topoisomerase I inhibitor isoliquiritigenin on HCC tumor cell. Biochem. Biophys. Res. Commun..

[51] Wang N., Wang Z., Wang Y., Xie X., Shen J., Peng C., You J., Peng F., Tang H., Guan X. (2015). Dietary compound isoliquiritigenin prevents mammary carcinogenesis by inhibiting breast cancer stem cells through WIF1 demethylation. Oncotarget.

[52] Kong L.D., Zhang Y., Pan X., Tan R.X., Cheng C.H.K. (2000). Inhibition of xanthine oxidase by liquiritigenin and isoliquiritigenin isolated from Sinofranchetia chinensis. Cell. Mol. Life Sci..

[53] Wu C.-H., Chen H.-Y., Wang C.-W., Shieh T.-M., Huang T.-C., Lin L.-C., Wang K.-L., Hsia S.-M. (2016). Isoliquiritigenin induces apoptosis and autophagy and inhibits endometrial cancer growth in mice. Oncotarget.

[54] Na A.-Y., Yang E.-J., Jeon J.M., Ki S.H., Song K.-S., Lee S. (2018). Protective Effect of Isoliquiritigenin against Ethanol-Induced Hepatic Steatosis by Regulating the SIRT1-AMPK Pathway. Toxicol. Res..

[55] Younas M., Hano C., Giglioli-Guivarc’H N., Abbasi B.H. (2018). Mechanistic evaluation of phytochemicals in breast cancer remedy: Current understanding and future perspectives. RSC Adv..

[56] Wang Z., Wang N., Liu P., Chen Q., Situ H., Xie T., Zhang J., Peng C., Lin Y., Chen J. (2014). MicroRNA-25 regulates chemoresistance-associated autophagy in breast cancer cells, a process modulated by the natural autophagy inducer isoliquiritigenin. Oncotarget.

[57] Wang K.-L., Hsia S.-M., Chan C.-J., Chang F.-Y., Huang C.-Y., Bau D.-T., Wang P.S. (2013). Inhibitory effects of isoliquiritigenin on the migration and invasion of human breast cancer cells. Expert Opin. Ther. Targets.

[58] Kwon H.M., Choi Y.J., Choi J.S., Kang S.W., Bae J.Y., Kang I.J., Jun J.G., Lee S.S., Lim S.S., Kang Y.H. (2007). Blockade of cytokine-induced endothelial cell adhesion molecule expression by licorice isoliquiritigenin through NF-kappaB signal disruption. Exp. Biol. Med..

[59] Zhang X., Yeung E.D., Wang J., Panzhinskiy E.E., Tong C., Li W., Li J. (2010). Isoliquiritigenin, a natural anti-oxidant, selectively inhibits the proliferation of prostate cancer cells. Clin. Exp. Pharmacol. Physiol..

[60] Hu F.-W., Yu C.-C., Hsieh P.-L., Liao Y.-W., Lu M.-Y., Chu P.-M. (2017). Targeting oral cancer stemness and chemoresistance by isoliquiritigenin-mediated GRP78 regulation. Oncotarget.

[61] Maggiolini M., Statti G., Vivacqua A., Gabriele S., Rago V., Loizzo M., Menichini F., Andò S. (2002). Estrogenic and antiproliferative activities of isoliquiritigenin in MCF7 breast cancer cells. J. Steroid Biochem. Mol. Biol..

[62] Anemone A., Consolino L., Conti L., Reineri F., Cavallo F., Aime S., Longo D.L. (2017). In vivo evaluation of tumour acidosis for assessing the early metabolic response and onset of resistance to dichloroacetate by using magnetic resonance pH imaging. Int. J. Oncol..

[63] Lau G.T.Y., Ye L., Leung L.K. (2009). The Licorice Flavonoid Isoliquiritigenin Suppresses Phorbol Ester-induced Cyclooxygenase-2 Expression in the Non-tumorigenic MCF-10A Breast Cell Line. Planta Med..

[64] Zheng H., Li Y., Wang Y., Zhao H., Zhang J., Chai H., Tang T., Yue J., Guo A.M., Yang J. (2014). Downregulation of COX-2 and CYP 4A signaling by isoliquiritigenin inhibits human breast cancer metastasis through preventing anoikis resistance, migration and invasion. Toxicol. Appl. Pharmacol..

[65] Ning S., Mu J., Shen Z., Zhu D., Jiang F., Wang X., Li Y. (2016). Isoliquiritigenin attenuates the invasive capacity of breast cancer cells via up-regulating the tumor suppressor RECK. RSC Adv..

[66] Li Y., Zhao H., Wang Y., Zheng H., Yu W., Chai H., Zhang J., Falck J.R., Guo A.M., Yue J. (2013). Isoliquiritigenin induces growth inhibition and apoptosis through downregulating arachidonic acid metabolic network and the deactivation of PI3K/Akt in human breast cancer. Toxicol. Appl. Pharmacol..

[67] Peng F., Tang H., Liu P., Shen J., Guan X.-Y., Xie X., Gao J., Xiong L., Jiangang S., Chen J. (2017). Isoliquiritigenin modulates miR-374a/PTEN/Akt axis to suppress breast cancer tumorigenesis and metastasis. Sci. Rep..

[68] Ning S., Zhu D., Shen Z., Liu J., Liu Y., Chen J., Li Z. (2017). Isoliquiritigenin attenuates MiR-21 expression via induction of PIAS3 in breast cancer cells. RSC Adv..

[69] Lin P.-H., Chiang Y.-F., Shieh T.-M., Chen H.-Y., Shih C.-K., Wang T.-H., Wang K.-L., Huang T.-C., Hong Y.-H., Li S.-C. (2020). Dietary Compound Isoliquiritigenin, an Antioxidant from Licorice, Suppresses Triple-Negative Breast Tumor Growth via Apoptotic Death Program Activation in Cell and Xenograft Animal Models. Antioxidants.

[70] Ye L., Gho W.M., Chan F.L., Chen S., Leung L.K. (2009). Dietary administration of the licorice flavonoid isoliquiritigenin deters the growth of MCF-7 cells overexpressing aromatase. Int. J. Cancer.

[71] Zorko B.A., Pérez L.B., De Blanco E.J.C. (2010). Effects of ILTG on DAPK1 promoter methylation in colon and leukemia cancer cell lines. Anticancer. Res..

[72] Yoshida T., Horinaka M., Takara M., Tsuchihashi M., Mukai N., Wakada M., Sakai T. (2008). Combination of isoliquiritigenin and tumor necrosis factor-related apoptosis-inducing ligand induces apoptosis in colon cancer HT29 cells. Environ. Health Prev. Med..

[73] Jin H., Lee S.H., Lee S.H. (2018). Isoliquiritigenin-mediated p62/SQSTM1 induction regulates apoptotic potential through attenuation of caspase-8 activation in colorectal cancer cells. Eur. J. Pharmacol..

[74] Auyeung K.K.-W., Auyeung J.K.K.A.K.K. (2009). Novel herbal flavonoids promote apoptosis but differentially induce cell cycle arrest in human colon cancer cell. Investig. New Drugs.

[75] Lee C.K., Son S.H., Park K.K., Park J.H.Y., Lim S.S., Chung W.-Y. (2008). Isoliquiritigenin Inhibits Tumor Growth and Protects the Kidney and Liver Against Chemotherapy-Induced Toxicity in a Mouse Xenograft Model of Colon Carcinoma. J. Pharmacol. Sci..

[76] Takahashi T., Takasuka N., Iigo M., Baba M., Nishino H., Tsuda H., Okuyama T. (2004). Isoliquiritigenin, a flavonoid from licorice, reduces prostaglandin E2 and nitric oxide, causes apoptosis, and suppresses aberrant crypt foci development. Cancer Sci..

[77] Huang Y.-L., Wei F., Zhao K., Zhang Y., Wang D., Ya-Li H. (2017). Isoliquiritigenin inhibits colorectal cancer cells HCT-116 growth by suppressing the PI3K/AKT pathway. Open Life Sci..

[78] Sechet E., Telford E., Bonamy C., Sansonetti P.J., Sperandio B. (2018). Natural molecules induce and synergize to boost expression of the human antimicrobial peptide beta-defensin-3. Proc. Natl. Acad. Sci. USA.

[79] Chen C., Huang S., Chen C.-L., Su S.-B., Fang D.-D. (2019). Isoliquiritigenin Inhibits Ovarian Cancer Metastasis by Reversing Epithelial-to-Mesenchymal Transition. Molecules.

[80] Chen H.-Y., Huang T.-C., Shieh T.-M., Wu C.-H., Lin L.-C., Hsia S.-M. (2017). Isoliquiritigenin Induces Autophagy and Inhibits Ovarian Cancer Cell Growth. Int. J. Mol. Sci..

[81] Mahalingam S., Gao L., Eisner J., Helferich W.G., Flaws J.A. (2016). Effects of isoliquiritigenin on ovarian antral follicle growth and steroidogenesis. Reprod. Toxicol..

[82] Li N., Yang L., Deng X., Sun Y. (2018). Effects of isoliquiritigenin on ovarian cancer cells. OncoTargets Ther..

[83] Yuan X., Yu B., Wang Y., Jiang J., Liu L., Zhao H., Qi W., Zheng Q. (2013). Involvement of endoplasmic reticulum stress in isoliquiritigenin-induced SKOV-3 cell apoptosis. Recent Pat. Anti-Cancer Drug Discov..

[84] Lee J.-E., Hong E.-J., Nam H.-Y., Hwang M., Kim J.-H., Han B.-G., Jeon J.-P. (2012). Molecular signatures in response to Isoliquiritigenin in lymphoblastoid cell lines. Biochem. Biophys. Res. Commun..

[85] Li D., Wang Z., Chen H., Wang J., Zheng Q., Shang J., Li J. (2009). Isoliquiritigenin induces monocytic differentiation of HL-60 cells. Free Radic. Biol. Med..

[86] Liu Q., Lv H., Wen Z., Ci X., Peng L. (2017). Isoliquiritigenin activates nuclear factor erythroid-2 related factor 2 to suppress the NOD-Like receptor protein 3 inflammasome and inhibits the NF-kappaB pathway in macrophages and in acute lung injury. Front. Immunol..

[87] Traboulsi H., Cloutier A., Boyapelly K., Bonin M.-A., Marsault É., Cantin A.M., Richter M.V. (2015). The Flavonoid Isoliquiritigenin Reduces Lung Inflammation and Mouse Morbidity during Influenza Virus Infection. Antimicrob. Agents Chemother..

[88] Ho W., Zhou Y. (2013). Combination of liquiritin, isoliquiritin and isoliquirigenin induce apoptotic cell death through upregulating p53 and p21 in the A549 non-small cell lung cancer cells. Oncol. Rep..

[89] Liu B., Yang J., Wen Q., Li Y. (2008). Isoliquiritigenin, a flavonoid from licorice, relaxes guinea-pig tracheal smooth muscle in vitro and in vivo: Role of cGMP/PKG pathway. Eur. J. Pharmacol..

[90] Hsu Y.-L., Kuo P.-L., Chiang L.-C., Lin C.-C. (2004). Isoliquiritigenin inhibits the proliferation and induces the apoptosis of human non-small cell lung cancer a549 cells. Clin. Exp. Pharmacol. Physiol..

[91] Ii T., Satomi Y., Katoh D., Shimada J., Baba M., Okuyama T., Nishino H., Kitamura N. (2004). Induction of cell cycle arrest and p21CIP1/WAF1 expression in human lung cancer cells by isoliquiritigenin. Cancer Lett..

[92] Park S.-J., Song H.-Y., Youn H.-S. (2009). Suppression of the TRIF-dependent signaling pathway of toll-like receptors by isoliquiritigenin in RAW264.7 macrophages. Mol. Cells.

[93] Lee S.H., Kim J.Y., Seo G.S., Kim Y.-C., Sohn D.H. (2009). Isoliquiritigenin, from Dalbergia odorifera, up-regulates anti-inflammatory heme oxygenase-1 expression in RAW264.7 macrophages. Inflamm. Res..

[94] Chen H., Zhang B., Yao Y., Chen N., Chen X., Tian H., Wang Z., Zheng Q. (2012). NADPH Oxidase-Derived Reactive Oxygen Species Are Involved in the HL-60 Cell Monocytic Differentiation Induced by Isoliquiritigenin. Molecules.

[95] Chen H., Zhang B., Yuan X., Yao Y., Zhao H., Sun X., Zheng Q. (2013). Isoliquiritigenin-induced effects on Nrf2 mediated antioxidant defence in the HL-60 cell monocytic differentiation. Cell Biol. Int..

[96] Youns M., Fu Y.-J., Zu Y.-G., Kramer A., Konkimalla V.B., Radlwimmer B., Sültmann H., Efferth T. (2010). Sensitivity and resistance towards isoliquiritigenin, doxorubicin and methotrexate in T cell acute lymphoblastic leukaemia cell lines by pharmacogenomics. Arch. Pharmacol..

[97] Zu Y., Liu X., Fu Y.-J., Shi X., Wu N., Yao L., Efferth T. (2009). Cytotoxic Activity of Isoliquiritigenin towards CCRF-CEM Leukemia Cells and its Effect on DNA Damage. Planta Med..

[98] Yu H., Li H., Li Y., Li M., Chen G. (2017). Effect of isoliquiritigenin for the treatment of atopic dermatitis-like skin lesions in mice. Arch. Dermatol. Res..

[99] Xiang S., Chen H., Luo X.-J., An B., Wu W., Cao S., Ruan S., Wang Z., Weng L., Zhu H. (2018). Isoliquiritigenin suppresses human melanoma growth by targeting miR-301b/LRIG1 signaling. J. Exp. Clin. Cancer Res..

[100] Chen X., Yang M., Hao W., Han J., Ma J., Wang C., Sun S., Zheng Q. (2016). Differentiation-inducing and anti-proliferative activities of isoliquiritigenin and all-trans-retinoic acid on B16F0 melanoma cells: Mechanisms profiling by RNA-seq. Gene.

[101] Chen X.Y., Li D.F., Han J.C., Wang B., Dong Z.P., Yu L.N., Pan Z.H., Qu C.J., Chen Y., Sun S.G. (2017). Reprogramming induced by isoliquiritigenin diminishes melanoma cachexia through mTORC2-AKT-GSK3beta signaling. Oncotarget.

[102] Chen X., Ren H.-H., Wang D., Chen Y., Qu C.-J., Pan Z.-H., Liu X., Hao W., Xu W.-J., Wang K. (2019). Isoliquiritigenin Induces Mitochondrial Dysfunction and Apoptosis by Inhibiting mitoNEET in a Reactive Oxygen Species-Dependent Manner in A375 Human Melanoma Cells. Oxidative Med. Cell. Longev..

[103] Wang Y., Ma J., Yan X., Chen X., Si L., Liu Y., Han J., Hao W., Zheng Q. (2016). Isoliquiritigenin Inhibits Proliferation and Induces Apoptosis via Alleviating Hypoxia and Reducing Glycolysis in Mouse Melanoma B16F10 Cells. Recent Pat. Anti-Cancer Drug Discov..

[104] Chen X., Zhang B., Yuan X., Yang F., Liu J., Zhao H., Liu L., Wang Y., Wang Z., Zheng Q. (2012). Isoliquiritigenin-Induced Differentiation in Mouse Melanoma B16F0 Cell Line. Oxidative Med. Cell. Longev..

[105] Chen X., Wu Y., Jiang Y., Zhou Y., Wang Y., Yao Y., Yi C., Gou L., Yang J. (2012). Isoliquiritigenin inhibits the growth of multiple myeloma via blocking IL-6 signaling. J. Mol. Med..

[106] Lv J., Fu Y., Cao Y., Jiang S., Yang Y., Song G., Yun C., Gao R. (2020). Isoliquiritigenin inhibits melanogenesis, melanocyte dendricity and melanosome transport by regulating ERK-mediated MITF degradation. Exp. Dermatol..

[107] Iwashita K., Kobori M., Yamaki K., Tsushida T. (2000). Flavonoids Inhibit Cell Growth and Induce Apoptosis in B16 Melanoma 4A5 Cells. Biosci. Biotechnol. Biochem..

[108] Huang Y., Liu C., Zeng W.-C., Xu G.-Y., Wu J.-M., Li Z.-W., Huang X.-Y., Lin R., Shi X. (2020). Isoliquiritigenin inhibits the proliferation, migration and metastasis of Hep3B cells via suppressing cyclin D1 and PI3K/AKT pathway. Biosci. Rep..

[109] Wang J.R., Luo Y.H., Piao X.J., Zhang Y., Feng Y.C., Li J.Q., Xu W.T., Zhang Y., Zhang T., Wang S.N. (2019). Mechanisms underlying isoliquiritigenin-induced apoptosis and cell cycle arrest via ROS-mediated MAPK/STAT3/NF-kappaB pathways in human hepatocellular carcinoma cells. Drug Dev. Res..

[110] Hsu Y.-L., Kuo P.-L., Lin C.-C. (2005). Isoliquiritigenin induces apoptosis and cell cycle arrest through p53-dependent pathway in Hep G2 cells. Life Sci..

[111] Hsu Y.-L., Kuo P.-L., Lin L.-T., Lin C.-C. (2005). Isoliquiritigenin Inhibits Cell Proliferation and Induces Apoptosis in Human Hepatoma Cells. Planta Med..

[112] Jang D.S., Park E.J., Kang Y.H., Hawthorne M.E., Vigo J.S., Graham J.G., Cabieses F., Fong H.H., Mehta R.G., Pezzuto J.M. (2003). Potential cncer chemopreventive flavonoids from the stems of Tephrosia toxicaria. J. Nat. Prod..

[113] Fang S.-C., Hsu C.-L., Lin H.-T., Yen G.-C. (2010). Anticancer Effects of Flavonoid Derivatives Isolated from Millettia reticulata Benth in SK-Hep-1 Human Hepatocellular Carcinoma Cells. J. Agric. Food Chem..

[114] Zhang B., Lai Y., Li Y., Shu N., Wang Z., Wang Y., Li Y., Chen Z. (2018). Antineoplastic activity of isoliquiritigenin, a chalcone compound, in androgen-independent human prostate cancer cells linked to G2/M cell cycle arrest and cell apoptosis. Eur. J. Pharmacol..

[115] Lee Y.M., Lim D.Y., Choi H.J., Jung J.I., Chung W.-Y., Park J.H.Y. (2009). Induction of Cell Cycle Arrest in Prostate Cancer Cells by the Dietary Compound Isoliquiritigenin. J. Med. Food.

[116] Kwon G.T., Cho H.J., Chung W.-Y., Park K.-K., Moon A., Park J.H.Y. (2009). Isoliquiritigenin inhibits migration and invasion of prostate cancer cells: Possible mediation by decreased JNK/AP-1 signaling. J. Nutr. Biochem..

[117] Jung J.I., Chung E., Seon M.R., Shin H.-K., Kim E.J., Lim S.S., Chung W.-Y., Park K.-K., Park J.H.Y. (2006). Isoliquiritigenin (ISL) inhibits ErbB3 signaling in prostate cancer cells. BioFactors.

[118] Jung J.I., Lim S.S., Choi H.J., Shin H.-K., Kim E.J., Chung W.-Y., Park K.-K., Park J.H.Y. (2006). Isoliquiritigenin induces apoptosis by depolarizing mitochondrial membranes in prostate cancer cells. J. Nutr. Biochem..

[119] Kanazawa M., Satomi Y., Mizutani Y., Ukimura O., Kawauchi A., Sakai T., Baba M., Okuyama T., Nishino H., Miki T. (2003). Isoliquiritigenin Inhibits the Growth of Prostate Cancer. Eur. Urol..

[120] Hirchaud F., Hermetet F., Ablise M., Fauconnet S., Vuitton D.A., Prétet J.-L., Mougin C. (2013). Isoliquiritigenin Induces Caspase-Dependent Apoptosis via Downregulation of HPV16 E6 Expression in Cervical Cancer Ca Ski Cells. Planta Med..

[121] Yuan X., Zhang B., Gan L., Wang Z.H., Yu B.C., Liu L.L., Zheng Q., Wang Z.P. (2013). Involvement of the mitochondrion-dependent and the endoplasmic reticulum stress-signaling pathways in isoliquiritigenin-induced apoptosis of HeLa cell. Biomed. Environ. Sci..

[122] Hsu Y.-L., Chia C.-C., Chen P.-J., Huang S.-E., Huang S.-C., Kuo P.-L. (2009). Shallot and licorice constituent isoliquiritigenin arrests cell cycle progression and induces apoptosis through the induction of ATM/p53 and initiation of the mitochondrial system in human cervical carcinoma HeLa cells. Mol. Nutr. Food Res..

[123] Zhang X., Wang S., Sun W., Wei C. (2018). Isoliquiritigenin inhibits proliferation and metastasis of MKN28 gastric cancer cells by suppressing the PI3K/AKT/mTOR signaling pathway. Mol. Med. Rep..

[124] Lee H.H., Lee S., Shin Y.-S., Cho M., Kang H.J., Cho H. (2016). Anti-Cancer Effect of Quercetin in Xenograft Models with EBV-Associated Human Gastric Carcinoma. Molecules.

[125] Ma J., Fu N.-Y., Pang D.-B., Wu W.-Y., Xu A.-L. (2001). Apoptosis Induced by Isoliquiritigenin in Human Gastric Cancer MGC-803 Cells. Planta Med..

[126] Kim D.-C., Ramachandran S., Baek S.-H., Kwon S.-H., Kwon K.-Y., Cha S.-D., Bae I., Cho C.-H. (2008). Induction of Growth Inhibition and Apoptosis in Human Uterine Leiomyoma Cells by Isoliquiritigenin. Reprod. Sci..

[127] Chen J., Liu C., Yang Q.-Q., Ma R.-B., Ke Y., Dong F., Wu X.-E. (2018). Isoliquiritigenin Suppresses Osteosarcoma U2OS Cell Proliferation and Invasion by Regulating the PI3K/Akt Signalling Pathway. Chemotherapy.

[128] Li C., Zhou X., Sun C., Liu X., Shi X., Wu S. (2019). Isoliquiritigenin inhibits the proliferation, apoptosis and migration of osteosarcoma cells. Oncol. Rep..

[129] Alshangiti A.M., Togher K.L., Hegarty S.V., Sullivan A.M., O’Keeffe G.W. (2019). The dietary flavonoid isoliquiritigenin is a potent cytotoxin for human neuroblastoma cells. Neuronal Signal..

[130] Zhao S., Chang H., Ma P., Gao G., Jin C., Zhao X., Zhou W., Jin B. (2015). Inhibitory effect of DNA topoisomerase inhibitor isoliquiritigenin on the growth of glioma cells. Int. J. Clin. Exp. Pathol..

[131] Yang H.-H., Zhang C., Lai S.-H., Zeng C.-C., Liu Y., Wang X.-Z. (2017). Isoliquiritigenin Induces Cytotoxicity in PC-12 Cells In Vitro. Appl. Biochem. Biotechnol..

[132] Si L., Yang X., Yanming W., Wang Y., Zheng Q. (2017). Isoliquiritigenin induces apoptosis of human bladder cancer T24 cells via a cyclin-dependent kinase-independent mechanism. Oncol. Lett..

[133] Drevon D., Fursa S.R., Malcolm A.L. (2016). Intercoder Reliability and Validity of WebPlotDigitizer in Extracting Graphed Data. Behav. Modif..

[134] Baba M., Asano R., Takigami I., Takahashi T., Ohmura M., Okada Y., Sugimoto H., Arika T., Nishino H., Okuyama T. (2002). Studies on Cancer Chemoprevention by Traditional Folk Medicines XXV.—Inhibitory Effect of Isoliquiritigenin on Azoxymethane-Induced Murine Colon Aberrant Crypt Focus Formation and Carcinogenesis. Biol. Pharm. Bull..

[135] Choi Y.H., Bae J.-K., Chae H.-S., Nhoek P., Choi J.-S., Chin Y.-W., Choi Y.O. (2016). Isoliquiritigenin ameliorates dextran sulfate sodium-induced colitis through the inhibition of MAPK pathway. Int. Immunopharmacol..

[136] Gao Y., Lv X., Yang H., Peng L., Ci X. (2020). Isoliquiritigenin exerts antioxidative and anti-inflammatory effects via activating the KEAP-1/Nrf2 pathway and inhibiting the NF-kappaB and NLRP3 pathways in carrageenan-induced pleurisy. Food Funct..

[137] Yu D., Liu X., Zhang G., Ming Z., Wang T. (2018). Isoliquiritigenin inhibits cigarette smoke-induced COPD by attenuating inflammation and oxidative stress via the regulation of the Nrf2 and NF-kappaB signaling pathways. Front. Pharmacol..

[138] Zhang W., Wang G., Zhou S. (2018). Protective effects of isoliquiritigenin on LPS-induced acute lung injury by activating PPAR-gamma. Inflammation.

[139] Yamazaki S., Morita T., Endo H., Hamamoto T., Baba M., Joichi Y., Kaneko S., Okada Y., Okuyama T., Nishino H. (2002). Isoliquiritigenin suppresses pulmonary metastasis of mouse renal cell carcinoma. Cancer Lett..

[140] Sun C., Wang Z.-H., Liu X.-X., Yang L.-N., Wang Y., Liu Y., Mao A.-H., Liu Y.-Y., Zhou X., Di C.-X. (2015). Disturbance of redox status enhances radiosensitivity of hepatocellular carcinoma. Am. J. Cancer Res..

[141] Park I., Park K.-K., Park J.H.Y., Chung W.-Y. (2009). Isoliquiritigenin induces G2 and M phase arrest by inducing DNA damage and by inhibiting the metaphase/anaphase transition. Cancer Lett..

[142] Lin L.-C., Wu C.-H., Shieh T.-M., Chen H.-Y., Huang T.-C., Hsia S.-M. (2017). The licorice dietary component isoliquiritigenin chemosensitizes human uterine sarcoma cells to doxorubicin and inhibits cell growth by inducing apoptosis and autophagy via inhibition of m-TOR signaling. J. Funct. Foods.

[143] Sun C., Zhang H., Ma X.-F., Zhou X., Gan L., Liu Y.-Y., Wang Z.-H. (2012). Isoliquiritigenin Enhances Radiosensitivity of HepG2 Cells via Disturbance of Redox Status. Cell Biophys..

[144] Yuan X., Zhang B., Chen N., Chen X., Liu L.-L., Zheng Q., Wang Z.-P. (2012). Isoliquiritigenin treatment induces apoptosis by increasing intracellular ROS levels in HeLa cells. J. Asian Nat. Prod. Res..

[145] Zhao H., Yuan X., Li D., Chen H., Jiang J., Wang Z., Sun X., Zheng Q. (2013). Isoliquiritigen Enhances the Antitumour Activity and Decreases the Genotoxic Effect of Cyclophosphamide. Molecules.

[146] Lin M., Wu D., Huang Y. (2016). Natural compounds ursolic acid and isoliquiritigenin target GRP78 to enhance human gastric cancer cell chemosensitivity by 5-fluorouracil. FASEB J..

[147] Yang N., Patil S., Zhuge J., Wen M.C., Bolleddula J., Doddaga S., Goldfarb J., Sampson H.A., Li X.M. (2013). Glycyrrhiza uralensis flavonoids present in anti-asthma formula, ASHMI, inhibit memory Th2 responses in vitro and in vivo. Phytother. Res..

[148] Moreno-Londoño A.P., Bello-Alvarez C., Pedraza-Chaverri J. (2017). Isoliquiritigenin pretreatment attenuates cisplatin induced proximal tubular cells (LLC-PK1) death and enhances the toxicity induced by this drug in bladder cancer T24 cell line. Food Chem. Toxicol..

[149] Gómez-Sierra T., Medina-Campos O.N., Solano J.D., Ibarra-Rubio M.E., Pedraza-Chaverri J. (2020). Isoliquiritigenin Pretreatment Induces Endoplasmic Reticulum Stress-Mediated Hormesis and Attenuates Cisplatin-Induced Oxidative Stress and Damage in LLC-PK1 Cells. Molecules.

[150] Siegel R.L., Mph K.D.M., Sauer A.G., Fedewa S.A., Butterly L.F., Anderson J.C., Cercek A., Smith R.A., Jemal A. (2020). Colorectal cancer statistics, 2020. Cancer J. Clin..

[151] Zhao M., Liu Q., Gong Y., Xu X., Zhang C., Liu X., Zhang C., Guo H., Zhang X., Gong Y. (2016). GSH-dependent antioxidant defense contributes to the acclimation of colon cancer cells to acidic microenvironment. Cell Cycle.

[152] Dai L., Pan G., Liu X., Huang J., Jiang Z., Zhu X., Gan X., Xu Q., Tan N. (2018). High expression of ALDOA and DDX5 are associated with poor prognosis in human colorectal cancer. Cancer Manag. Res..

[153] Wang G., Wang Y.-Z., Yu Y., Wang J.-J., Yin P.-H., Xu K. (2019). Triterpenoids Extracted from Rhus chinensis Mill Act Against Colorectal Cancer by Inhibiting Enzymes in Glycolysis and Glutaminolysis: Network Analysis and Experimental Validation. Nutr. Cancer.

[154] Jayson G.C., Kohn E.C., Kitchener H.C., Ledermann J.A. (2014). Ovarian cancer. Lancet.

[155] Nasim F., Sabath B.F., Eapen G.A. (2019). Lung Cancer. Med. Clin. N. Am..

[156] Siegel R.L., Miller K.D.M., Jemal A. (2018). Cancer statistics. Cancer J. Clin..

[157] Romaszko A.M., Doboszyńska A. (2018). Multiple primary lung cancer: A literature review. Adv. Clin. Exp. Med..

[158] Tian T., Sun J., Wang J., Liu Y., Liu H. (2018). Isoliquiritigenin inhibits cell proliferation and migration through the PI3K/AKT signaling pathway in A549 lung cancer cells. Oncol. Lett..

[159] Hsia S.-M., Yu C.-C., Shih Y.-H., Chen M.Y., Wang T.-H., Huang Y.-T., Shieh T.-M. (2015). Isoliquiritigenin as a cause of DNA damage and inhibitor of ataxia-telangiectasia mutated expression leading to G2/M phase arrest and apoptosis in oral squamous cell carcinoma. Head Neck.

